# Extracellular vesicles from hiPSC-NSCs can prevent peripheral inflammation-induced cognitive dysfunction with inflammasome inhibition and improved neurogenesis in the hippocampus

**DOI:** 10.1186/s12974-023-02971-y

**Published:** 2023-12-12

**Authors:** Gunel Ayyubova, Maheedhar Kodali, Raghavendra Upadhya, Leelavathi N. Madhu, Sahithi Attaluri, Yogish Somayaji, Bing Shuai, Shama Rao, Goutham Shankar, Ashok K. Shetty

**Affiliations:** Institute for Regenerative Medicine, Department of Cell Biology and Genetics, School of Medicine, Texas A&M Health Science Center, 1114 TAMU, 206 Olsen Boulevard, College Station, TX 77843 USA

**Keywords:** Activated microglia, Cognitive dysfunction, Extracellular vesicles, Human neural stem cells, Human pluripotent stem cells, NLRP3 inflammasomes

## Abstract

Extracellular vesicles (EVs) released by human induced pluripotent stem cell-derived neural stem cells (hiPSC-NSCs) are enriched with miRNAs and proteins capable of mediating robust antiinflammatory activity. The lack of tumorigenic and immunogenic properties and ability to permeate the entire brain to incorporate into microglia following intranasal (IN) administrations makes them an attractive biologic for curtailing chronic neuroinflammation in neurodegenerative disorders. We tested the hypothesis that IN administrations of hiPSC-NSC-EVs can alleviate chronic neuroinflammation and cognitive impairments induced by the peripheral lipopolysaccharide (LPS) challenge. Adult male, C57BL/6J mice received intraperitoneal injections of LPS (0.75 mg/kg) for seven consecutive days. Then, the mice received either vehicle (VEH) or hiPSC-NSC-EVs (~ 10 × 10^9^ EVs/administration, thrice over 6 days). A month later, mice in all groups were investigated for cognitive function with behavioral tests and euthanized for histological and biochemical studies. Mice receiving VEH after LPS displayed deficits in associative recognition memory, temporal pattern processing, and pattern separation. Such impairments were associated with an increased incidence of activated microglia presenting NOD-, LRR-, and pyrin domain containing 3 (NLRP3) inflammasomes, elevated levels of NLRP3 inflammasome mediators and end products, and decreased neurogenesis in the hippocampus. In contrast, the various cognitive measures in mice receiving hiPSC-NSC-EVs after LPS were closer to naive mice. Significantly, these mice displayed diminished microglial activation, NLRP3 inflammasomes, proinflammatory cytokines, and a level of neurogenesis matching age-matched naïve controls. Thus, IN administrations of hiPSC-NSC-EVs are an efficacious approach to reducing chronic neuroinflammation-induced cognitive impairments.

**Supplementary Information:**

The online version contains supplementary material available at 10.1186/s12974-023-02971-y.

## Introduction

Neuroinflammation is one of the hallmarks of many neuropsychiatric and neurodegenerative conditions, including Alzheimer’s disease (AD), Parkinson’s disease, Huntington’s disease, multiple sclerosis, and autism spectrum disorders [1–4]. Therefore, testing therapeutic strategies in animal models exhibiting acute and chronic neuroinflammation, such as those induced through lipopolysaccharide (LPS), has considerable significance. LPS comprises primary cell wall constituents of Gram-negative bacteria, which can act as pathogen-associated molecular patterns (PAMPs) recognized by the innate immune system and induce immune response. Since LPS is a natural ligand for toll-like receptor 4 (TLR4), its administration activates TLR signaling in microglia, the resident immune cells in the central nervous system leading to long-term neuroinflammation [5]. While microglial activation in the initial phase of neuroinflammation mediates tissue homeostasis and neuroprotection, their continued activation leads to chronic neuroinflammation, which promotes synapse loss and neurodegeneration via the incessant production of proinflammatory cytokines and reactive oxygen species [6, 7]. Persistent systemic inflammation can also cause microglia-mediated chronic neuroinflammation typified by increased TLR4 signaling, activation of NOD-, LRR- and pyrin domain containing 3 (NLRP3) inflammasomes and the complement system, synaptotoxicity, extracellular amyloid-beta (Aβ) accumulation, neurodegeneration, and reduced hippocampal neurogenesis [8–10].

Learning, memory, and several behavioral impairments have been reported in models of peripheral inflammation with similarities to AD [11–13]. Therefore, LPS administrations have been widely employed in animal models of neuroinflammation to evaluate test compounds or biologics to alleviate cognitive decline associated with AD [14–16]. Accumulation of amyloid plaques in the extracellular space and abnormally phosphorylated tau within neurons have been recognized as the major pathological hallmarks linked to cognitive decline in AD [17, 18]. However, amyloid-plaque-reducing therapies have not been able to slow down the progression of AD significantly [19, 20]. Such failure has led to a closer examination of microglial dysfunction and the resulting chronic neuroinflammation as a potential target for slowing down AD progression [21]. Increasingly, neuroinflammation is not just considered as a secondary response to Aβ accumulation and tau pathology, but also as a causative factor in the etiology of AD [22]. Several studies imply that neuroinflammatory conditions develop much before the onset of clinical symptoms of AD, which has led to the hypothesis that neuroinflammation drives the disease progression independently of the Aβ plaques and neurofibrillary tangles, and interaction of these three factors exacerbates the disease progression [23]. Moreover, based on the "infection hypothesis” of AD, neuroinflammation transpiring from infectious agents and their products, has received significant interest [24, 25]. For example, bacterial infections have been correlated with AD development, as the plasma and/or brains of AD patients and animal models display 3–6 times higher levels of LPS compared to healthy controls [25, 26]. LPS has also been seen in amyloid plaques of AD brains, particularly in the perinuclear region of neurons [27, 28]. Collectively, there is enough evidence to support the notion that AD-like pathogenesis could emerge from significant systemic inflammation. In this context, rigorous long-term studies in LPS-based animal models could provide insights on the role of neuroinflammation in AD. Such models could also be used to test novel therapeutic strategies focused on modulating neuroinflammation in AD.

The precise mechanisms by which systemic inflammation triggers the onset and progression of AD are yet to be ascertained. Therapies modulating NLRP3 inflammasome activation in the brain could be one of the ways to restrain neuroinflammation and the progression of neuropathological changes and cognitive decline in AD. A few studies have suggested that the administration of mesenchymal stem cells (MSCs) or extracellular vesicles (EVs) released by MSCs can modulate neuroinflammation in LPS models [29, 30]. However, no studies have tested the efficacy of neural stem cell (NSC)-derived EVs in LPS models hitherto. Recently, we have shown that EVs released by human induced pluripotent stem cell (hiPSC)-derived NSCs are enriched with miRNAs and proteins capable of mediating antiinflammatory, anti-apoptotic, antioxidant, and neurogenic effects [31, 32]. NSC-derived EVs also contain miRNAs and proteins capable of promoting synaptogenesis, neuroprotection, neural plasticity, and cognitive function [32, 33]. Furthermore, the lack of tumorigenic and immunogenic properties and the competence to quickly permeate the entire brain to incorporate into neurons and microglia following intranasal (IN) administrations [31, 33, 34] makes them an attractive biologic for restraining chronic neuroinflammation in neurodegenerative disorders. Therefore, the current study examined whether IN administration of hiPSC-NSC-EVs in the early stage of LPS-induced systemic inflammation would prevent brain dysfunction. The results provide a novel evidence that IN administration of an optimal dose of hiPSC-NSC-EVs is an effective approach for preventing LPS-induced neuroinflammatory sequelae associated with enduring impairments in cognition, memory and social interaction. Notably, such functional effects were associated with inhibiting signaling events that perpetuate neuroinflammation, reduced synapse loss, and preservation of hippocampal neurogenesis.

## Materials and methods

### Animals

Adult male C57BL/6J mice (*n* = 67) purchased from Jackson Laboratories (Jackson Labs, Bar Harbor, ME, USA) were employed. The animal care and experimental procedures were conducted per the animal protocol approved by the Animal Care and the Use Committee (IACUC) of Texas A&M University School of Medicine. Mice were group-housed (*n* = 4–5/cage) in an environmentally controlled room with ad libitum food and water and 12-h light and 12-h dark cycles.

### Experimental design

**Fig. 1:**
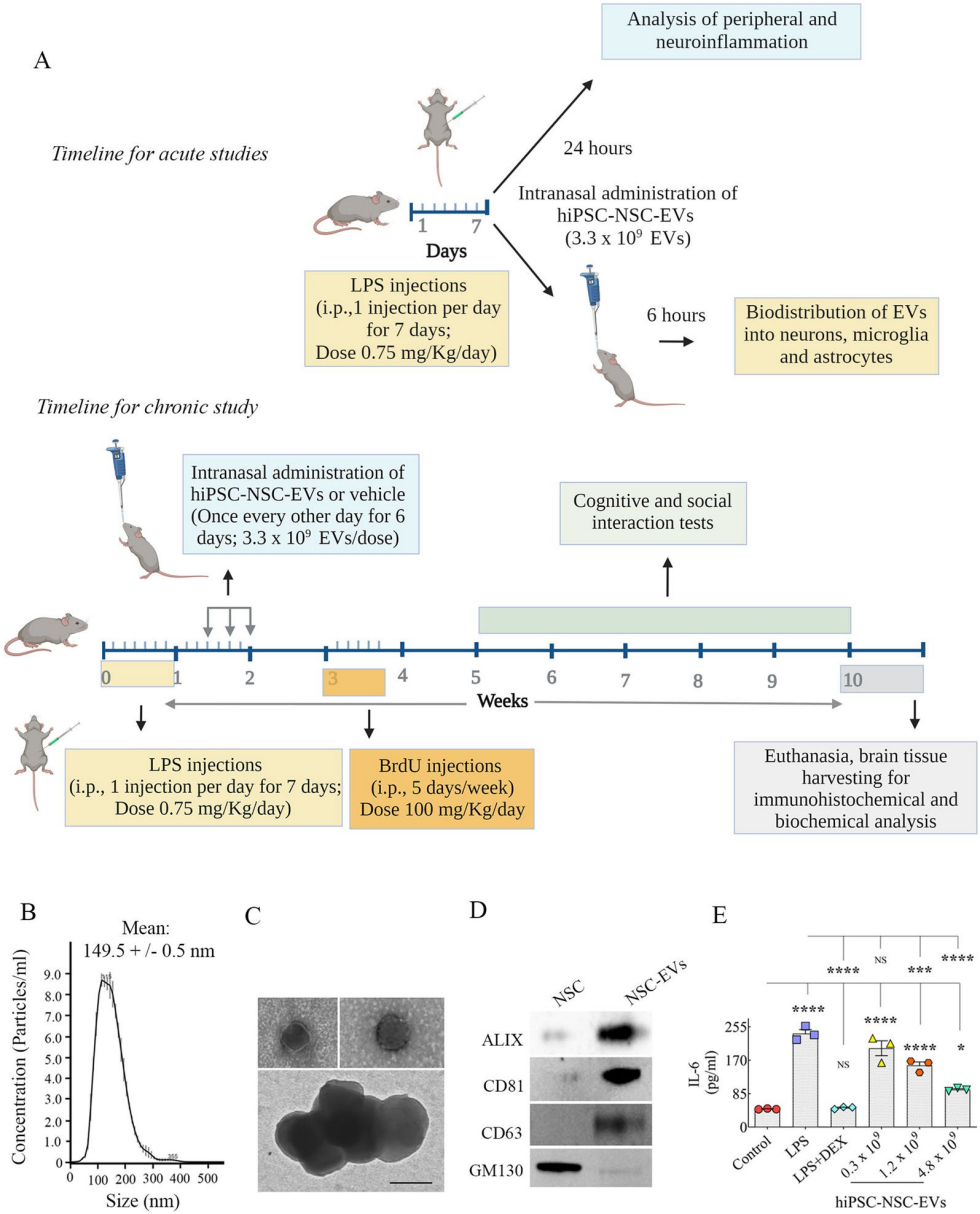
Timeline of animal experiments and characterization of extracellular vesicles (EVs) from human induced pluripotent stem cell-derived neural stem cells (hiPSC-NSCs). **A** Illustrates the timeline of acute and chronic in vivo studies in mice, depicting seven LPS injections in week 1, intranasal administrations of vehicle or hiPSC-NSC-EVs in week 2, analysis of peripheral and neuroinflammation at 24 h post-LPS, characterization of the distribution of intranasally administered EVs in the brain at 6 h post-administration, BrdU injections in week 4 (once daily for five days), behavioral tests in weeks 6–10, followed by euthanasia and brain tissue collection for immunohistochemical and biochemical analyses. Graph **B** shows the concentration and size of hiPSC-NSC-EVs obtained through NanoSight tracking analysis. The images in **C** display the morphology and size of hiPSC-NSC-EVs revealed through transmission electron microscopy. Scale bar, 100 nm. The blots in **D** demonstrate the presence of EV-specific proteins ALIX, CD81, and CD63 and the absence of the Golgi complex protein GM130 in hiPSC-NSC-EVs. Graph E shows the dose-dependent reductions in IL-6 production by the different doses of hiPSC-NSC-EV treatment. *, *p* < 0.05; ***, *p* < 0.001; and ****, *p* < 0.0001; NS not significant

A cartoon depicting the experimental design and timelines of different experiments performed is available in Fig. 1A. Following two weeks of acclimatization, eight-week-old male C57BL/6J mice were assigned randomly to the naïve control, LPS + Veh, or LPS + EV groups (n = 15/group). The mice in LPS + Veh and LPS + EVs groups received daily intraperitoneal injections of LPS (*Escherichia coli* serotype: 0111:B4; Sigma–Aldrich, St. Louis, MO, USA) dissolved in normal saline at a dose of 0.75 mg/kg for seven consecutive days (days 1–7), as described in a previous study [13]. Animals were kept warm after each administration. Seven days of LPS administration employed in the study has displayed upregulation of multiple proinflammatory markers in the serum and the brain, including interleukin-1 beta (IL-1β), tumor necrosis factor-alpha (TNFα), and prostaglandin E2. The model also exhibits downregulation of IL-4 and IL-10 in the circulating blood and the brain [13]. Starting from day 8, these mice received three IN doses of either PBS (LPS + Veh group) or hiPSC-NSC-EVs (~ 3.3 × 10^9^ EVs/dose, LPS + EVs group) over 6 days, with each dose separated by two days. Thus, peripheral and central inflammations were present during hiPSC-NSC-EV administration. EV administrations were done as described in our previous reports [32, 35, 36]. Altogether, each mouse received ~ 10 × 10^9^ EVs. One week after the final EV administration, the animals received five intraperitoneal injections of 5ʹ-bromodeoxyuridine (BrdU) for 5 days (100 mg/Kg) to measure the extent of net hippocampal neurogenesis. Three weeks later, mice in all groups were investigated for cognitive and memory function with a battery of behavioral tests, followed by euthanasia and collection of brain tissues at 9 weeks post-EV administration to analyze neuroinflammation and neurogenesis.

### hiPSC-derived NSC cultures, isolation and characterization of EVs

The protocols for generating NSCs from hiPSCs, isolation of NSC-derived EVs, and characterization of the number, size, and markers of EVs are detailed in the previous reports [31]. After culturing the hiPSC colonies (IMR90-4; Wisconsin International Stem Cell Bank, Madison, WI, USA) for 24 h, the medium was replaced with a neural induction medium to transform hiPSCs into NSCs. The NSC cultures were passaged every seven days, and NSCs from different passages were cryoprotected and stored in liquid nitrogen. For the isolation of NSC-derived EVs, frozen vials containing passage 11 NSCs were grown till they reached 70% confluency, followed by seeding them into 150 × 20 mm diameter culture plates in an NSC expansion medium. Once the cells attained ~ 90% confluency, the spent media was harvested and stored at − 80 ℃ for further EV isolation. The purity of P11 NSCs was confirmed through immunofluorescence staining for NSC-specific markers such as nestin (anti-nestin, 1:1000; EMD Millipore, Burlington, MA, USA) and Sox-2 (anti-Sox-2, 1:300; Santacruz Biotechnology, Dallas, TX, USA). NSC-derived EVs were isolated via centrifugation, filtration, sequential application of anion exchange, and size-exclusion chromatography protocols [31]. Our earlier study has reported the characterization of the antiinflammatory and neurogenic properties of hiPSC-NSC-EVs and their miRNA and protein composition [31]. Nanoparticle tracking analysis with NanoSight was used to determine the concentration and size of EVs. Western blotting was used to investigate the EV-specific markers, apoptosis-linked 2 interacting protein X (ALIX), CD81 and CD63, and the deep cellular marker GM130 in hiPSC-NSC-EV preparations [31].

### Characterization of hiPSC-NSC-EVs for size, number, and expression of ALIX, CD81, CD63 and GM130

Nanoparticle tracking analysis was used to determine the concentration and size of EVs, as detailed in our previous studies [31, 32]. Using western blotting, we verified the expression of EV-specific markers such as CD63, CD81, and ALIX and the lack of GM130 (a Golgi complex protein not expressed in EVs). In brief, an aliquot of EVs (100 µl) was mixed with 100 µl of a mammalian protein extractor reagent (Thermo Fischer Scientific, Waltham, MA, USA) and lysed as described previously [37]. The total protein concentration in the lysate was determined using the Pierce BCA protein assay kit (ThermoFisher Scientific), and 40 μg of protein was loaded and separated using 4–12% NuPAGE Bis–Tris Gels (ThermoFisher Scientific). Next, proteins were transferred onto nitrocellulose membranes using Thermo Fisher Scientific's iBlot2 gel transfer technology. Then the membrane was processed for protein detection using antibodies against CD63 (1:1000 BD Biosciences, San Jose, California, CA, USA), CD81 (1:1000 BD Biosciences), and ALIX (1:1000, Santa Cruz, Santa Cruz, Dallas, TX, USA). The signal was then detected using an ibright 1500 (Invitrogen, Waltham, MA, USA) and an excellent chemiluminescence substrate kit (ThermoFisher). In order to rule out the contamination of EV lysate with deep cellular proteins, the protein-separated membrane was processed for GM130 expression (1:1000; Santa Cruz) compared with the hiPSC-NSC lysate [31]. LPS-stimulated murine macrophage test was used to investigate the antiinflammatory activity of hiPSC-NSC-EVs [31, 32].

### Visualization of hiPSC-NSC EVs through transmission electron microscopy (TEM)

Our previous studies have described the protocol employed for TEM imaging [31, 35]. Briefly, 5 μl of the EV suspension diluted in PBS was placed at room temperature on 300 mesh carbon-coated copper grids (Electron Microscopy Sciences, Hatfield, PA, USA). After 5 minutes, the excess liquid in the grids was blotted with filter paper and rinsed twice with distilled water. The grids were stained by continuous dripping in 0.5% uranyl acetate (150 μl) solution at 45° inclination. The excess liquid was removed, and the grids were air-dried at room temperature for 10 min. The images were taken using an FEI Morgagni 268 transmission electron microscope with a MegaView III CCD camera. hNSC-EV diameters were computed using the “Analyze” tool in the Image J software as an average of the measured diameters along four axes (x, y, x + 45°, y + 45°).

### Characterization of antiinflammatory activity of hiPSC-NSC-EVs using macrophage assay

The isolated hiPSC-NSC-EVs were tested for their antiinflammatory activity using LPS-stimulated mouse macrophages, as described in our previous reports [31, 32]. Briefly, freshly thawed murine monocyte/macrophage (RAW 264.7; ATCC, Manassas, VA, USA) stocks were cultured overnight in 48-well plates at 100,000 cells/well density. Next, the adhered cells were treated with 10 ng/ml LPS (Sigma, St Louis, MO, USA), LPS plus dexamethasone (DEX, 1 µg/ml, Sigma), or LPS plus various doses of hiPSC-NSC-EVs (0.3, 1.2, or 4.8 × 10^9^ EVs) for 4 h. Then the conditioned media were collected, and the concentration of interleukin 6 (IL6) in the medium was measured using ELISA (R&D Systems, Minneapolis, MN, USA). Only the batches of hiPSC-NSC-EVs significantly reducing IL6 release from LPS-stimulated macrophages were administered to mice.

### Analysis of the extent of systemic inflammation and neuroinflammation in LPS-treated mice at the time of hiPSC-NSC-EV administration

A smaller cohort of mice from naive and LPS groups (*n* = 6/group) was euthanized, and serum, liver, and brain (hippocampus) samples were harvested a day after the last LPS injection to ascertain the extent of LPS-induced systemic inflammation and neuroinflammation at the time of hiPSC-NSC-EV administration. Blood was collected from these mice, and serum was stored at − 80 ℃. Fresh liver tissues were harvested, snap-frozen, and stored at − 80 ℃ until further use. Fresh brains were dissected and stored at − 80 ℃, and hippocampi were dissected before performing biochemical assays. For biochemical assays, a portion of liver tissue and hippocampal tissues were lysed by sonication in a tissue extraction buffer containing a protease inhibitor and phosphatase inhibitor (ThermoFisher Scientific) for 20 s at 4 ℃. The lysed solution was centrifuged at 15,000 g for 10 min, and the supernatant was aliquoted and kept at -80°C until needed. We measured the concentration of proinflammatory cytokines TNFα, IL-1β, IL-6, and IL-18. The ELISA Kits were from R&D Systems (Minneapolis, MN, USA).

### Analysis of biodistribution of IN-administered PKH26-labeled hiPSC-NSC-EVs in LPS-treated mice

To examine the biodistribution of IN-administered hiPSC-NSC-EVs, a smaller cohort of LPS-treated mice (*n* = 4) received IN administration of PKH26-labeled hiPSC-NSC-EVs (4 × 10^9^) a day after the last LPS injection and perfused with 4% paraformaldehyde 6 h later. The brain tissues were processed, and every 15th 30-µm section through the entire brain was collected and processed for immunofluorescence to visualize neuron-specific nuclear antigen-positive (NeuN +) neurons, ionized calcium-binding adaptor protein 1-positive (IBA-1 +) microglia, and glial fibrillary acidic protein-positive (GFAP +) astrocytes. The primary antibodies comprised anti-chicken NeuN (1:1000, Millipore Sigma, St. Louis, MO, USA), goat anti-IBA-1 (1:1000, Abcam, Cambridge, MA, USA), and rabbit anti-GFAP (1:2000, Millipore) followed by incubation in donkey anti-chicken IgG with Alexa Fluor 488 (1:200, Invitrogen), donkey anti-goat IgG with Alexa Fluor 488 (1:200, Invitrogen) and donkey anti-rabbit IgG with Alexa Fluor 488 (1:200, Invitrogen), respectively. Z-section analysis in a confocal microscope was employed to localize the association of PKH26 + hiPSC-NSC-EVs in various neural cell types in various regions of the forebrain, midbrain, and hindbrain.

### Behavioral tests

The behavioral tests commenced 3 weeks after EV or vehicle treatment and comprised temporal pattern processing, associative recognition memory, pattern separation, and social interaction tasks. In each test, Any-maze software tracked the behavioral patterns of mice. In all tests, animals exploring objects for ≥ 20 s were included for statistical comparisons, as described in our previous reports [35, 36].

### Assessment of associative recognition memory

**Fig. 2:**
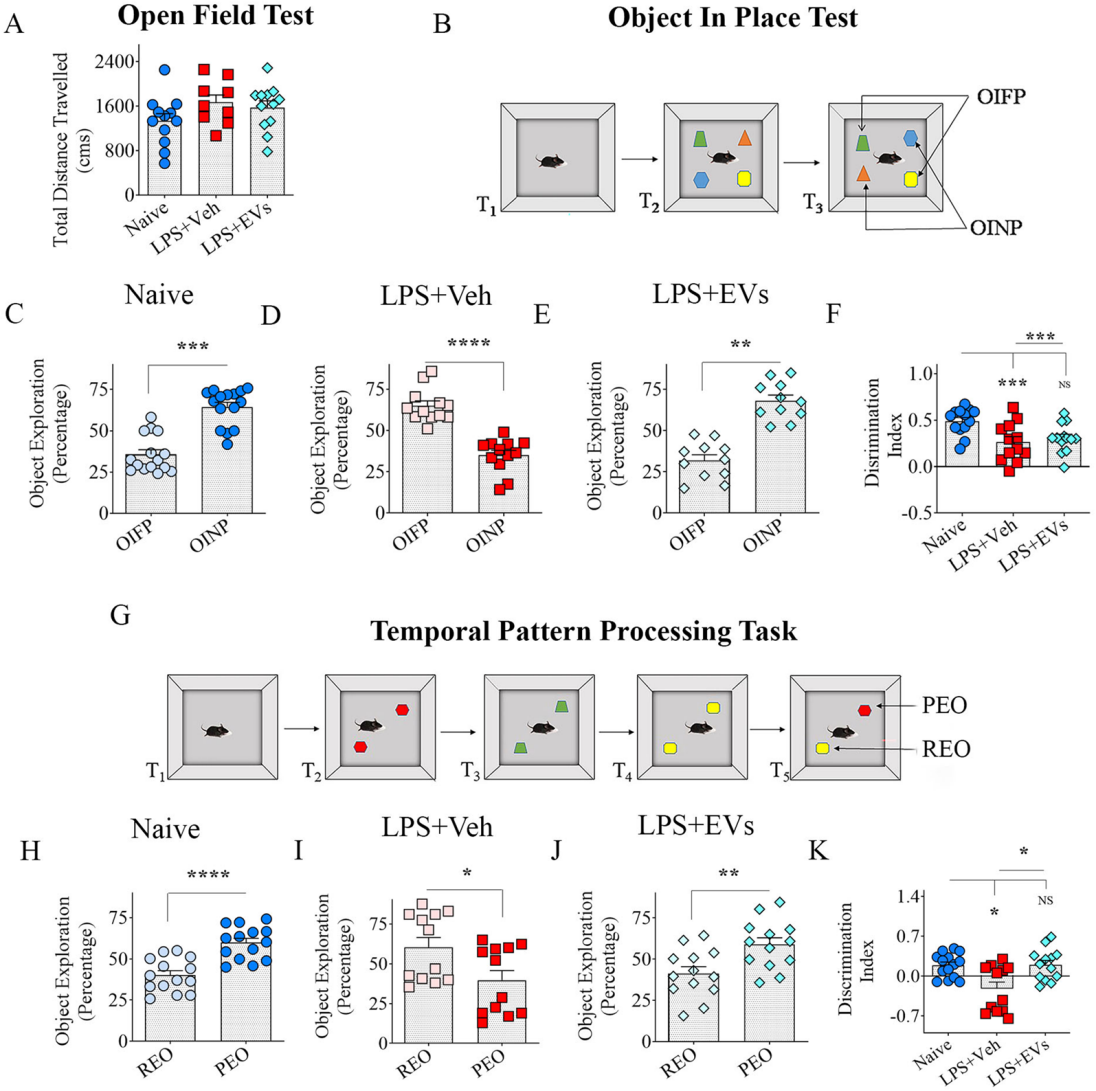
Intranasal administration of extracellular vesicles (EVs) from human induced pluripotent stem cell-derived neural stem cells (hiPSC-NSCs) prevented lipopolysaccharide (LPS)-induced impairments in associative recognition memory formation and temporal pattern processing. Bar chart **A** compares total distances traveled in an open field test between naïve control, LPS + Veh, and different LPS + EV groups. The cartoon in **B** shows the various trials (T1–T3) involved in the object-in-place test. The bar charts in **C**–**E** compare the propensity of animals to explore objects in novel and familiar places (OINP and OIFP), whereas bar chart F compares the OINP discrimination index across the three groups. The cartoon in **G** illustrates the different trials (T1–T5) involved in the temporal pattern processing task. The bar charts in **H**–**J** compare the preference of animals to explore the previously explored object (PEO) versus the recently explored object (REO), whereas bar chart **K** compares the PEO discrimination index across the three groups. *, *p* < 0.05; **, *p* < 0.01; ***, *p* < 0.001; and ****, *p* < 0.0001; *NS* not significant

Associative recognition memory function was assessed through an object-in-place test (OIPT). The associative recognition memory is a prerequisite to recognizing objects in the context where animals encountered them, as well as the information on the things and locations. OIPT involves neural activity in several regions, including the medial prefrontal cortex, hippocampus, and perirhinal cortex, involving multiple neural circuits [38]. Animals from different groups (*n* = 12–15/group) underwent three trials in OIPT. A cartoon depicting the sequence of trials (T1–T3) is shown in Fig. 2A. In the acquisition phase, each animal was placed in the center of an empty arena for 5 min (T1). After an inter-trial interval (ITI) of 5 min, the animal was placed in the same arena with four distinct objects placed in four quadrants for 5 min (T2). After an ITI of 30 min, the animal was placed in the arena again with two objects swapping their locations diagonally (T3). The objects swapping the locations were considered objects in novel places (OINP), whereas those maintaining locations were considered objects in familiar places (OIFP). Times spent exploring the OINP vis-a-vis times spent with the OIFP were compared within groups. In addition, the total object exploration times (TOETs) in T2 and T3 and the OINP discrimination index (DI) were computed and compared across groups [37].

### Evaluation of the temporal pattern processing

Temporal pattern processing (TPP) is vital for various intelligent behaviors like hearing, vision, speech, and music. TPP depends mainly on the integrity of the hippocampal CA1 subfield [39–41]. This function requires encoding patterns over time to recognize and generate temporal patterns. All animals from different groups (*n* = 12–15/group) were tested for their proficiency in TPP. A cartoon depicting the sequence of trials (T1–T5) is shown in Fig. 2F. Following acclimatization to the open field apparatus for 5 min (T1), the animals were allowed to explore three distinct pairs of identical objects sequentially in the sample phase (T2–T4, 5 min of object exploration separated by an ITI of 30 min). Then, two distinct objects, comprising one previously explored object (PEO) and another recently explored object (REO), were simultaneously presented (T5, 5 min). Times spent with the PEO vis-a-vis REO were collected and compared. The PEO-DI was also measured and compared across groups.

### Appraisal of the hippocampus-associated pattern separation function

The proficiency of animals for distinguishing similar but not identical experiences into non-overlapping representations in the hippocampus, a pattern separation test (PST) was employed. Pattern separation depends on the integrity of the dentate gyrus (DG) and the extent of neurogenesis [42, 43]. Animals from different groups (*n* = 12–15/group) were tested in 4 trials (Fig. 2A), as detailed in our previous reports [35, 44]. T1–T2 involved the acclimatization of the animal to the open field apparatus (T1) and the exploration of a pair of identical objects in the same arena but placed on floor pattern 1 (T2). After an ITI of 30 min, the animal explored a second pair of identical objects placed on floor pattern 2 (T3). Thirty minutes later, in T4, the animal explored one of the objects from T3, and an object from T2, placed on pattern 2 (familiar and novel objects on pattern 2, or FO and NO on P2). Times spent with the NO on P2 versus FO on P2 were collected and compared. The DI for NO on P2 was also measured and compared across groups.

### Assessment of social interaction

**Fig. 3:**
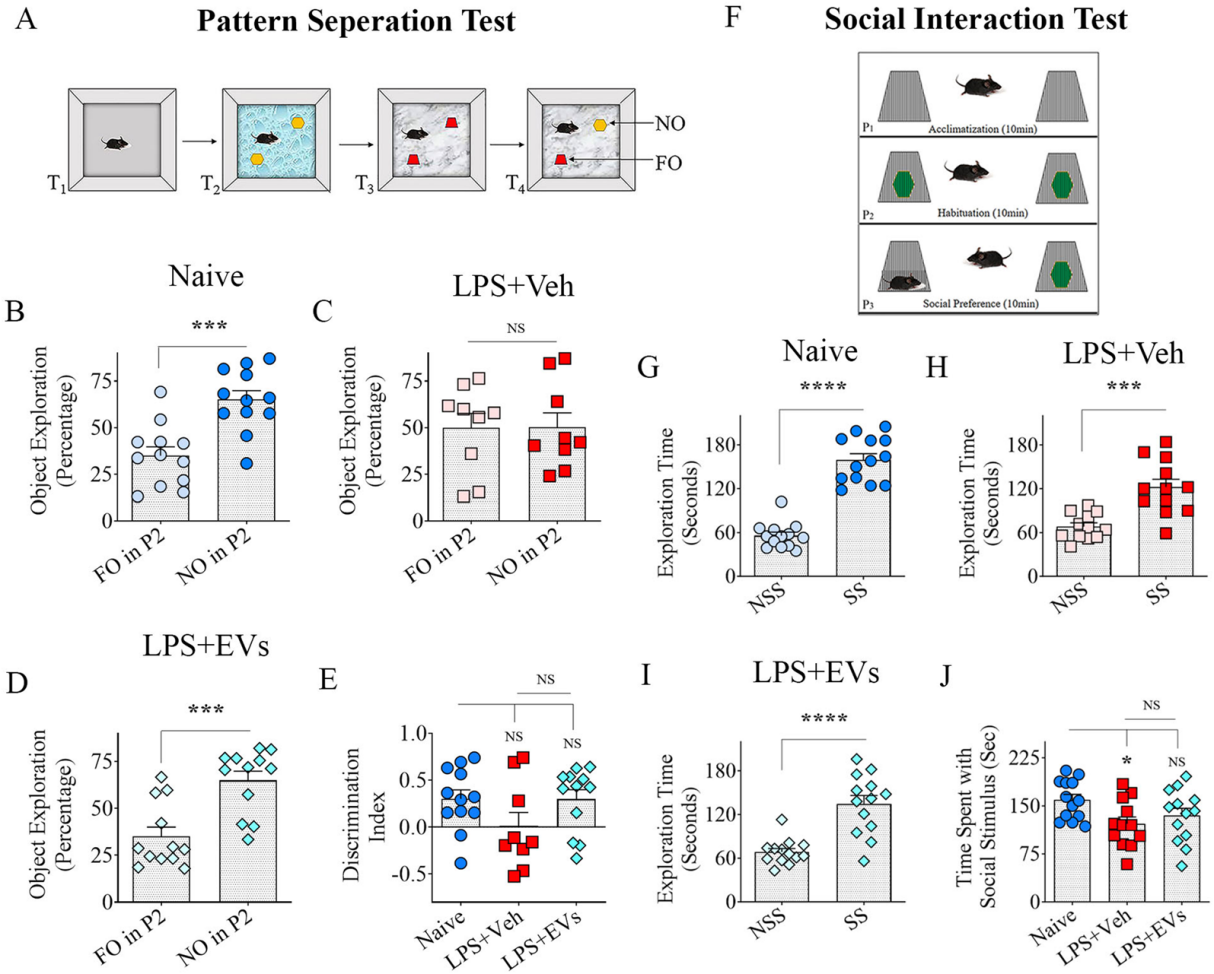
Intranasal administration of extracellular vesicles (EVs) from human induced pluripotent stem cell-derived neural stem cells (hiPSC-NSCs) eased lipopolysaccharide (LPS)-induced pattern separation deficits and improved social interaction behavior. The cartoon in **A** shows the various trials (T1–T4) involved in a pattern separation test. The bar charts in B-D compare the propensity of animals to explore the novel object in P2 (NO in P2) vis-à-vis the familiar object in P2 (FO in P2), whereas bar chart E compares the NO in P2 discrimination index across the three groups. The cartoon in F shows three phases (P1–P3) in a social interaction test. The bar charts in **B**–**D** compare the propensity of animals to explore the social stimulus (SS) vis-à-vis the nonsocial stimulus (NSS). The bar chart in J compares time spent with the social stimulus for the 10-min duration across the three groups. *, *p* < 0.05; ***, *p* < 0.001; and ****, *p* < 0.0001; *NS* not significant

The preference for interacting with a social stimulus over a nonsocial stimulus was investigated under conditions of equal salience using a social interaction test (Fig. 3H, 45]. Sixty minutes after habituating the animal to an open field apparatus for 10 min (T1), the animal was allowed to explore a pair of identical objects placed within the inverted grill mesh holders for 10 min to familiarize the animal with the objects placed under the holders (pre-test phase, T2). Sixty minutes later, in T3, one of the objects within the grill mesh is replaced with an age- and sex-matched wild-type mouse. The mouse became the social stimulus, whereas the object maintained within the second grill mesh served as the nonsocial stimulus in this trial. The grill mesh, made of sturdy steel and measuring 10.2 × 10.8 cm in diameter with mesh bars separated by 2–3 cm gaps, allowed adequate interaction and sniffing between the test animal and the animal placed within the gill mesh but prevented any fighting between them. Times spent by the test animal interacting with social and nonsocial stimuli are computed and compared to evaluate its preference. These included times spent in sniffing the grill mesh or directly engaging with the social stimulus.

### Euthanasia, tissue processing, immunohistochemistry and ELISA

Following behavioral tests, subgroups of animals in each group (n=7–8/group) were perfused with 4% paraformaldehyde, and the brain tissues were processed for cryostat sectioning. Thirty-micrometer-thick coronal sections were collected serially and stored at − 20 ℃ in a cryobuffer until further use [46, 47]. These sections were used for immunohistochemical and immunofluorescence staining (*n* = 7–8/group). Additional subgroups of animals (*n* = 7–8/group) were deeply anesthetized, and fresh brain tissues were harvested following decapitation. The tissues were snap-frozen and stored at − 80 ℃ until further use. For biochemical assays, microdissected hippocampal tissues were lysed by sonication using a sonic dismembrator (Sigma Aldrich Corp) and a tissue extraction buffer (Invitrogen) containing a protease inhibitor (Sigma-Aldrich Corp. St. Louis, MO) for 30 s at 4 ℃. The lysed solution was centrifuged at 15,000 g for 10 min, and the supernatant was aliquoted and kept at − 80 ℃ until needed. The protein concentration in various samples was quantified using the Pierce BCA reagent kit (Thermo Fisher Scientific). Hippocampal tissue lysates were employed for measuring the concentrations of mediators and endproducts of NLRP3 signaling, including the nuclear fraction of the nuclear factor-κB (NF-kBp65), NLRP3, apoptosis-associated speck-like protein containing a caspase recruitment domain (ASC) and cleaved caspase-1 and proinflammatory cytokines IL-1β and IL-18 using ELISA [36].

Our previous studies have detailed immunohistochemical methods employed in this study [35, 48, 49]. In each animal, every 15th or 20th section through the whole hippocampus was processed for immunohistochemical identification of IBA-1 + microglia, GFAP + astrocytes, BrdU-positive newly born cells and doublecortin (DCX) positive newly generated neurons. The primary antibodies comprised goat anti-IBA-1 (1:1000, Abcam. Cambridge, MA), rabbit anti-GFAP (1:3000, Agilent Tech, Carpinteria, CA), anti-rat BrdU (1:200, Abcam), and a goat anti-DCX (1:300, Abcam). The secondary antibodies comprised horse biotinylated anti-rabbit, anti-rat or anti-goat IgG (1:250, Vector Labs, Burlingame, CA). The avidin–biotin complex reagent and the chromogen Vector gray were purchased from Vector Labs. The sections were mounted on gelatin-coated slides, counterstained with nuclear fast red (Vector Labs), dehydrated, cleared, coverslipped with permount, and examined with a Nikon E600 microscope.

### Quantification of microglia, astrocytes, and newly born neurons

The numbers of IBA-1 + microglia and GFAP + astrocytes per unit volume (per 0.1 mm3) were quantified using three serial sections through the mid-region of the hippocampus in each animal (*n* = 5–6/group). The total number of newly born DCX + neurons in the subgranular zone-granular cell layer (SGZ-GCL) of the hippocampus (*n* = 5/group) was quantified using every 15th section through the entire hippocampus. All cell counts employed the optical fractionator method in a StereoInvestigator system attached to a color digital video camera interfaced with a Nikon E600 microscope (Microbrightfield, Williston, Vermont, USA), as described in our previous reports [50–52]. Furthermore, the area fractions of GFAP immunoreactive astrocytes in the DG and hippocampal CA1 and CA3 subfields were evaluated by Image J (3 sections/subfield/animal, *n* = 6/group).

### Morphometric analysis of IBA-1 + microglia

The morphological complexity and the area occupied by IBA-1 + microglia were measured in every animal by tracing the soma and processes of individual microglia using the Neurolucida tracing system (Microbrightfield) linked to a Nikon microscope. Microglia from the dentate molecular layer and stratum radiatum of CA1 and CA3 subfields were chosen randomly. To ensure constant sampling, only microglia displaying evident staining of the entire cell, located predominantly in the middle third of the section's thickness, and processes that did not overlap with the processes of other cells were chosen. In each group, 36 microglia (9 cells/animal, four animals/group) were traced individually using an oil immersion 100X lens. The data, such as the average area occupied by individual cells, the average total process length, and the number of nodes and endings, were calculated and compared across groups. In addition, using the NeuroExplorer component of the Neurolucida program, Sholl’s concentric circle analysis was performed to measure the pattern and extent of processes at different distances from the soma.

### Quantification of activated microglia

All immunofluorescence studies employed representative sections (3–4 sections/animal). The sections were washed thoroughly in PBS, treated with the normal donkey serum for 30 min, and incubated in primary antibody solutions overnight. Activated microglia were visualized through a dual immunofluorescence protocol in which sections were first incubated in a cocktail of goat anti-IBA-1 (1:1000, Abcam) and rat anti-ED-1 (CD68; 1:500, Bio-Rad, Hercules, CA) followed by incubation in a cocktail of donkey anti-goat IgG with Alexa Fluor 488 (1:200, Invitrogen) and donkey anti-rat IgG with Alexa Flour 594 (1:200, Invitrogen). The percentages of microglia expressing IBA-1 and ED-1 were next quantified through Z-section analysis in a Nikon confocal microscope [31, 36].

### Measurement of NLRP3 inflammasome complex in microglia

A triple immunofluorescence protocol was employed for visualizing NLRP3, ASC, and IBA-1 in microglia. The sections were incubated in a cocktail containing goat anti-NLRP3 (1:500, Millipore, Burlington), mouse anti-ASC (1:500, Santa Cruz), and rabbit anti-IBA-1 (1:1000 Abcam). The secondary antibodies comprised donkey anti-goat IgG conjugated with Alexa Fluor 488 (1:200, Invitrogen), donkey anti-mouse conjugated with Alexa Fluor 594 (1:200, Invitrogen), and anti-donkey rabbit conjugated with Alexa Fluor 405 (1:200, Abcam). The sections were next probed through a 1-μm-thick, Z-section analysis using Leica THUNDER 3D Imager. The total number of NLRP3 inflammasomes (i.e., structures co-expressing both NLRP3 and ASC) per unit area (~ 216 μm2) in the CA1 and CA3 subfields were measured using two sections per animal (n = 6/group). In addition, the percentages of IBA-1 + microglia displaying NLRP3 inflammasomes (i.e., cells positive for IBA-1, NLRP3, and ASC) were computed [36].

### *Quantification of BrdU* + *newly born cells and DCX* + *newly born neurons*

Every 15th thirty-micrometer section through the entire hippocampus was used for quantifying the numbers of BrdU-positive newly born cells or DCX-positive newly born neurons in the SGZ-GCL of the hippocampus (*n* = 5 sections/marker/animal). A StereoInvestigator system (Microbrightfield, Williston, Vermont, USA) was employed to quantify BrdU + and DCX + cells using 100X objective lens and stereological methods, as detailed in the previous reports [50]. The numbers of BrdU + cells and DCX + cells quantified using StereoInvestigator represent absolute counts for the entire SGZ-GCL of the hippocampus.

### *Measurement of neuronal differentiation of BrdU* + *newly born cells, and quantification of net hippocampal neurogenesis*

We employed a Nikon confocal microscope to perform Z-section analyses to determine the percentage of BrdU + cells expressing NeuN in the SGZ-GCL. Three representative sections from the anterior, mid, and posterior levels of the hippocampus were processed for BrdU-NeuN dual immunofluorescence, as described in our previous studies [53]. In each animal, 25–30 BrdU + cells from three representative sections were examined for NeuN expression (*n* = 6/group). Subsequently, the numbers of new neurons born in the second week after LPS injections and survived for 7 weeks in the hippocampus of LPS mice receiving the vehicle or hIPSC-NSC-EVs and age-matched naive mice were determined using stereology and percentages of BrdU + cells expressing NeuN in the SGZ-GCL were determined using Z-section analysis in a confocal microscope [53]. Thus, extrapolating the absolute numbers of BrdU + cells with the respective percentages of BrdU + cells expressing NeuN facilitated quantifying the total number of mature neurons added to the GCL during 5 days in various groups.

### Quantification of synaptophysin (Syn) and post-synaptic density protein 95 (PSD95) in the hippocampus

We employed western blots of hippocampal tissue lysates to quantify Syn and PSD95, as described in our recently published study [48]. The proteins in the membranes were identified using antibodies against Syn (1:5000, ProteinTech, Rosemont, IL, USA), PSD95 (1:1000, Abcam), and β-tubulin (1:10,000, Abcam) after being transferred onto a nitrocellulose membrane using the iBlot2 gel transfer device (ThermoFisher Scientific). β-tubulin was employed as a loading control for hippocampal tissue lysates. The protein signals were then detected using the chemiluminescence reagents in the ECL detection kit (ThermoFisher Scientific) and visualized using the iBright imaging equipment (ThermoFisher Scientific). Image J software was used to normalize the intensity of each targeted band (Syn, 38 kDa; PSD-95: 95 kDa) to a comparable β-tubulin band (50 kDa), which was also run alongside Syn and PSD-95.

### Statistical analysis

Statistical analyses utilized Prism software 10.0.3. Comparisons between two datasets employed a two-tailed, unpaired Student’s *t*-test or Mann–Whitney *U* test when standard deviations differed significantly between the groups. Comparisons involving three or more datasets employed one-way analysis of variance (ANOVA) with Tukey's multiple comparison post hoc tests. When individual groups failed the normality test (Shapiro–Wilk test), we performed the non-parametric Kruskal–Wallis test, followed by Dunn’s post hoc tests. In all comparisons, *p* < 0.05 was considered a statistically significant value.

## Results

### hiPSC-NSC-EVs displayed EV-specific markers and typical EV morphology in TEM

NanoSight analysis of EVs isolated from P11 hiPSC-NSC cultures through anion-exchange and size-exclusion chromatographic methods revealed the presence of small EVs with a mean size of 149.5 µm (Fig. 1B). Moreover, TEM confirmed the presence of double-membrane small vesicles in EV preparations employed in this study (Fig. 1C). Furthermore, hiPSC-NSC-EVs expressed multiple EV-specific markers (Fig. 1D). These include tetraspanins CD63 and CD81, an accessory protein of the endosomal sorting complex required for transport (ESCRT) ALIX, (Fig. 1D). However, these EVs lacked the Golgi protein GM130, as detailed in our previous reports [31, 32].

### hiPSC-NSC-derived EVs suppressed the upregulation of IL-6 in LPS-stimulated macrophages

The antiinflammatory activity of hiPSC-NSC-EVs on LPS-stimulated murine macrophage cells was confirmed as described in our previous reports [31]. LPS alone significantly increased macrophage IL-6 release (*p* < 0.0001; Fig. 1E). However, DEX or higher dosages (1.2 or 4.8 × 10^9^) of hiPSC-NSC-EVs suppressed LPS-induced IL-6 production by macrophages (*p* < 0.001–0.0001; Fig. 1E). Maximal suppression was observed with a treatment of 4.8 × 10^9^ EVs (p < 0.0001 Fig. 1E). However, the lowest dosage of EVs (0.3 × 10^9^) failed to suppress the production of IL-6. Thus, dose-dependent suppression of IL-6 release revealed that hiPSC-NSC-derived EVs have robust antiinflammatory activity.

### Seven days of LPS treatment induced both systemic inflammation and neuroinflammation

We measured proinflammatory cytokines TNF-α, IL-1β, IL-6, and IL-18 in the liver, serum, and hippocampal tissue lysates collected a day after the last LPS injection. Such analysis revealed an upregulation of these proinflammatory cytokines in the liver, serum, and hippocampus of LPS-treated mice compared to naive mice (*p* < 0.05—0.001; Additional file 1: Fig. S2). These results confirmed that at the timepoint of hiPSC-NSC-EV administration, both systemic inflammation and neuroinflammation were present.

### IN-administered hiPSC-NSC-EVs incorporated into neurons and microglia and came in contact with astrocytes

When examined 6 h post-administration, IN-administered PKH26-labeled hiPSC-NSC-EVs were incorporated into the soma of NeuN + neurons and IBA-1 + microglia in LPS-treated mice (Additional file 1: Figs. 3, 4). They also came in contact with the plasma membranes of soma and processes of GFAP + astrocytes (Additional file 1: Fig. 5). Similar pattern was observed in virtually all regions of the forebrain, midbrain, and hindbrain. Such targeting is akin to our earlier observations for hiPSC-NSC-EVs in naive mice and rats following IN-administration (31).

### LPS-treated mice receiving vehicle or hiPSC-EVs did not display motor impairments

Before commencing specific behavioral tests, animals in all groups were evaluated for possible motor impairments using an open field test (OFT). One-ANOVA analysis with Tukey’s post hoc tests for total distances traveled across groups revealed that neither the LPS + Veh nor LPS + EVs groups significantly differed from the naïve control group (Fig. 2A). Thus, LPS-treated mice did not display motor impairments during behavioral tests evaluating cognitive and mood function.

### hiPSC-NSC-EV treatment after LPS averted associative recognition memory impairment

An OIPT investigated the proficiency of mice for associative recognition memory (Fig. 2A). Because the dependability of this task depends on conscientious exploration of the location of four distinct objects in T2 (Additional file 1: Fig. 6A), only animals that explored objects for at least 20 s in T2 were included for data analysis. Most animals in every group (11–15/group) met the ≥ 20 s TOET criterion for T2. Naive control mice displayed competence for making associations between objects and locations, which was evident from their propensity to explore the OINP in T3 (p < 0.001; Fig. 2C). Vehicle-treated LPS mice spent more time exploring the OIFP than OINP (*p* < 0.0001; Fig. 2D), but EV-treated LPS mice maintained a preference to explore the OINP over the OIFP (*p* < 0.01; Fig. 2E). The DI for the OINP significantly differed between the three groups (*p* < 0.0001; Fig. 2F). Post hoc tests revealed significantly reduced DI in vehicle-treated LPS mice compared to the naïve control mice (*p* < 0.001; Fig. 2F), but the DI was comparable between naïve mice and EV-treated LPS mice (*p* > 0.05). The TOETs diverged between groups in some trials (Additional file 1: Fig. S6A-B), but all mice included for data analysis in all groups explored objects for 20 or more seconds. Overall, systemic LPS administration impaired associative recognition memory function linked to the integrity of the medial prefrontal cortex, the hippocampus, and the perirhinal cortex, but IN administration of hiPSC-NSC-EVs after LPS treatment prevented such dysfunction.

### hiPSC-NSC-EV treatment after LPS prevented temporal pattern processing dysfunction

TPPT examined the ability of mice to recognize and generate temporal patterns by encoding patterns over time. Since the validity of this test depends on careful exploration of the location of three distinct pairs of identical objects in T2–T4 (Fig. 2G), only animals that explored objects for at least 20 s in T2–T4 were included for data analysis. Most animals in every group met this criterion (*n* = 12–14/group). While naïve control mice showed competency for TPP by spending more time exploring the PEO over REO (Fig. 2H, *p* < 0.0001), vehicle-treated LPS mice instead explored the REO for extended periods than the PEO (Fig. 2I; *p* < 0.05). In contrast, the behavior of hiPSC-NSC-EV-treated LPS mice was similar to naive control mice, as they preferred to explore the PEO more than the REO (Fig. 2J, p < 0.01). Furthermore, the DI for PEO varied significantly between groups, with post hoc tests revealing reduced DI in vehicle-treated LPS mice (Fig. 2K; *p* < 0.05) and normalized DI in EV-treated LPS mice compared to naive mice (Fig. 2K; *p* > 0.05). The TOETs varied between groups in some trials (Additional file 1: Fig. S6C–F), but all mice included for data analysis in all groups explored objects for 20 or more seconds. Thus, systemic LPS administration impaired a cognitive function linked to the integrity of the hippocampal CA1 subfield, but IN administration of hiPSC-NSC-EVs after LPS treatment prevented such dysfunction.

### hiPSC-NSC-EV treatment after LPS eased pattern separation dysfunction

PST evaluated the ability of animals to distinguish similar but not identical experiences by encoding similar representations in a non-overlapping manner in the hippocampus. Since the validity of this test depends on carefully exploring the location of two distinct pairs of objects placed on specific patterns in T2 and T3 (Fig. 3A), only animals that explored objects for at least 20 s in T2–T3 were included for data analysis. The majority of animals in every group met the criterion (*n* = 9–12/group). Naïve control mice showed proficiency for pattern separation, which was apparent from their increased tendency to explore the NO over the FO on P2 in T4 (*p* < 0.001; Fig. 3B). Vehicle-treated LPS mice exhibited impaired pattern separation, as they spent nearly similar amounts of their object exploration time with the NO and FO on P2 (*p* > 0.05; Fig. 3C). In contrast, EV-treated LPS mice maintained their competence for pattern separation, which was evident from their exploration of the NO of P2 for longer durations than the FO on P2 in T4 (*p* < 0.001, Fig. 3D). However, the DI for NO on P2 did not reveal statistically significant differences between groups (*p* > 0.05 Fig. 3E) suggesting that EV treatment only partially protected pattern separation function in LPS mice, which may be due to a lower ITI employed in the study. As noted in other tests, the TOETs varied between groups in some trials (Additional file 1: Fig. 1G, H), but all mice included for data analysis in all groups explored objects for 20 or more seconds. Thus, systemic LPS administration induced pattern separation dysfunction, but IN administration of hiPSC-NSC-EVs after LPS treatment curtailed such dysfunction.

### hiPSC-NSC-EV treatment after LPS maintained better social interaction behavior

Comparing the times spent by the test animal interacting with social stimulus (SS) vis-a-vis nonsocial stimulus (NSS) revealed a quantitative measure of social interaction in the vehicle or hiPSC-NSC-EV-treated LPS mice (Fig. 3F, *n* = 12–15/group). Comparing times spent with the NSS vis-à-vis the SS for the entire 10-min duration of the test for each group revealed that animals in all three groups (Naïve, LPS + Veh, LPS + EVs) showed higher predilection for the SS over the NSS (*p* < 0.001–0.0001, Fig. 3G–I). However, comparing times spent with the SS across groups revealed that the LPS + Veh group explored SS for significantly reduced time compared to the naïve group (*p* < 0.05, Fig. 3J). Conversely, the LPS + EVs group explored SS for similar periods as the naïve group (*p* > 0.05, Fig. 3J). However, times spent with the SS did not differ significantly between the LPS + Veh and LPS + EVs groups (*p* > 0.05, Fig. 3J). Thus, mice in the LPS + EVs group behaved like the naïve control group, whereas the behavior of mice in the LPS + Veh group differed from the naïve control group. Nonetheless, since within-group comparisons demonstrated an increased propensity to explore the SS in every group, it is clear that LPS treatment did not impair but moderately reduced their social interaction behavior. However, hiPSC-NSC-EV treatment after LPS maintained their social interaction behavior like naive control animals.

### hiPSC-NSC-EV treatment reduced the proliferation and proinflammatory phenotype of microglia

**Fig. 4:**
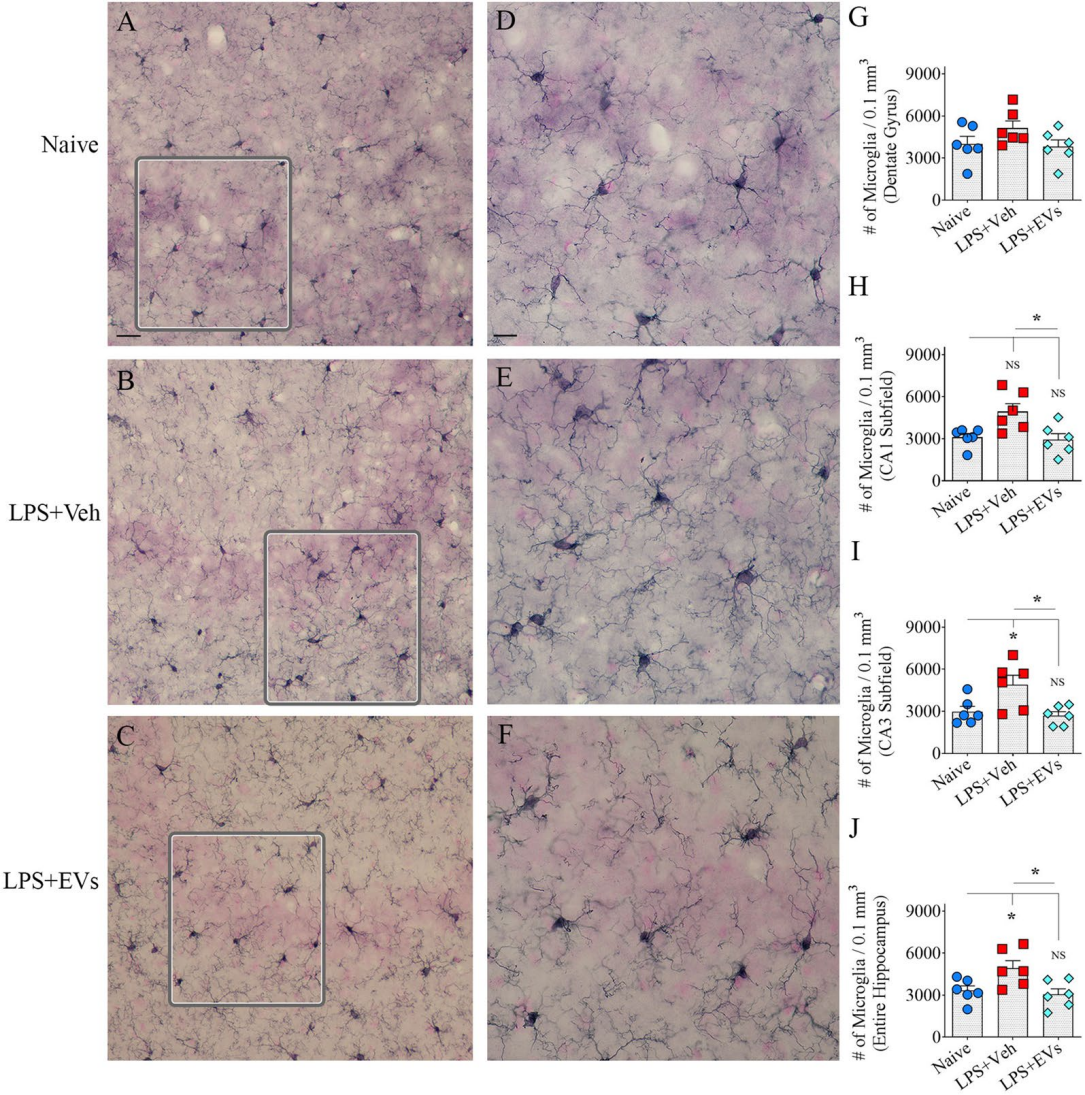
Intranasal administration of extracellular vesicles (EVs) from human induced pluripotent stem cell-derived neural stem cells (hiPSC-NSCs) restrained lipopolysaccharide (LPS)-induced microgliosis. Figures **A**–**F** Illustrate examples of the density of IBA1 + microglia in the CA3 subfield from naïve control (**A**, **D**), vehicle-treated LPS (LPS + Veh, **B**, **E**), extracellular vesicle-treated LPS (LPS + EVs, **C**, **F**) animal groups. The bar charts **G**-**J** compare the numbers of IBA1 + structures in the dentate gyrus (**G**), the CA1 subfield (**H**), the CA3 subfield (**I**), and the entire hippocampus (**J**) between different groups. Scale bar, **A**–**C** = 100 μm; **D**–**F** = 20 μm *, *p* < 0.05; *NS* not significant

**Fig. 5:**
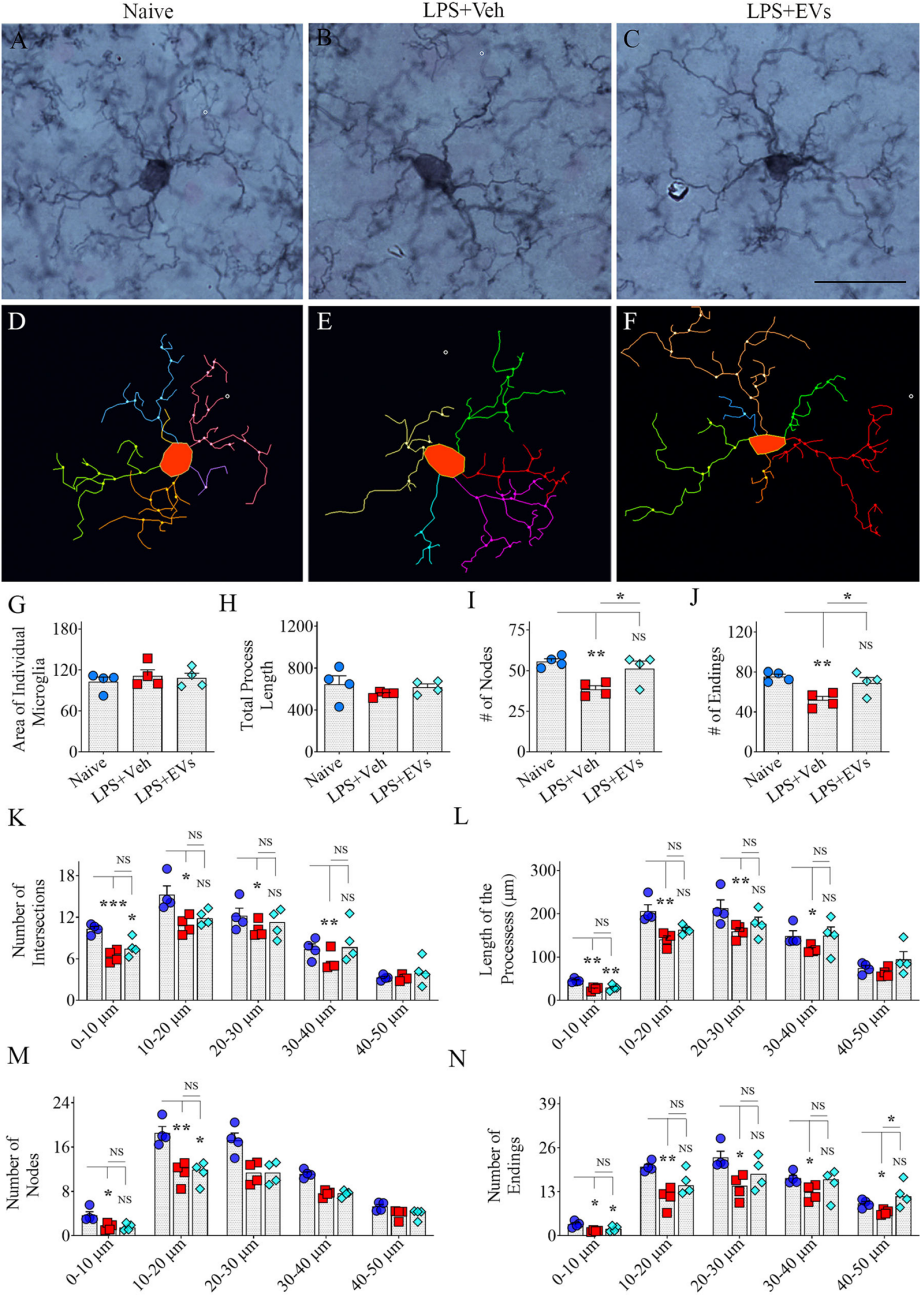
Intranasal administration of extracellular vesicles (EVs) from human induced pluripotent stem cell-derived neural stem cells (hiPSC-NSCs) reduced lipopolysaccharide (LPS)-induced transformation of microglia into proinflammatory phenotypes. Figures A-F illustrate representative examples of microglial morphology traced with Neurolucida in the CA3 subfield of hippocampus from naïve control (**A**, **D**), vehicle-treated LPS (LPS + Veh, **B**, **E**), and extracellular vesicle-treated LPS (LPS + EVs, **C**, **F**) animal groups. The bar charts G-J compare the average area occupied by individual microglia (**G**), the mean total process length (**H**), and the average number of nodes (**I**) and endings (**J**) in processes across groups. The bar charts K-N compare the number of intersections (**K**), total process length (**L**), and the number of nodes (**M**) and endings (**N**) in processes across groups at 0–10 μm, 10–20 μm, 20–30 μm, 30–40 μm, and 40–50 μm distances from the soma. Scale bar, **A**–**F** = 25 μm. *p < 0.05; **p < 0.01; and ***p < 0.001; *NS* not significant

The density and morphology of microglia varied between groups in different brain regions. Examples of the distribution of IBA-1 + microglia in the CA3 subfield from different groups are illustrated (Fig. 4A–F). Compared to naive mice, microglial numbers significantly increased in the CA1 and CA3 subfields, the entire hippocampus, of the vehicle-treated LPS mice (p < 0.05) but not in the EV-treated LPS mice (Fig. 4G–J). Representative examples of the morphology of individual IBA-1 + microglia in different groups are illustrated with tracings (Fig. 5A–F). Microglia in vehicle-treated LPS mice exhibited a proinflammatory phenotype with a reduced ramification of processes. In contrast, the morphology of microglia in EV-treated LPS mice mostly resembled noninflammatory phenotypes typically seen in naïve control mice by displaying extensive ramification of their processes (Fig. 5D–F). Morphometric analyses confirmed these observations (Fig. 5G–N). Compared to naïve control mice, microglia in vehicle-treated LPS mice displayed significantly reduced numbers of nodes and endings (*p* < 0.01, Fig. 5I, J). Furthermore, Sholl’s analysis revealed reductions in the numbers of intersections, lengths of processes, numbers of nodes, and endings at several distances from the soma in vehicle-treated LPS mice (*p* < 0.05–0.001, Fig. 5K–N). In contrast, a vast majority of morphometric measurements in EV-treated LPS mice matched microglia from naïve control mice (*p* > 0.05, Fig. 5G–N). The exceptions in vehicle-treated LPS mice compared to naïve control mice were reduced numbers of intersections, lengths of processes, and numbers of endings at 0–10 um distance from the soma (*p* < 0.05–0.01, Fig. 5K, L, N). Thus, hiPSC-NSC-EV treatment restrained the proliferation of microglia and the transformation of microglia into a proinflammatory phenotype.

### hiPSC-NSC-EV treatment reduced activated microglia presenting CD68

**Fig. 6:**
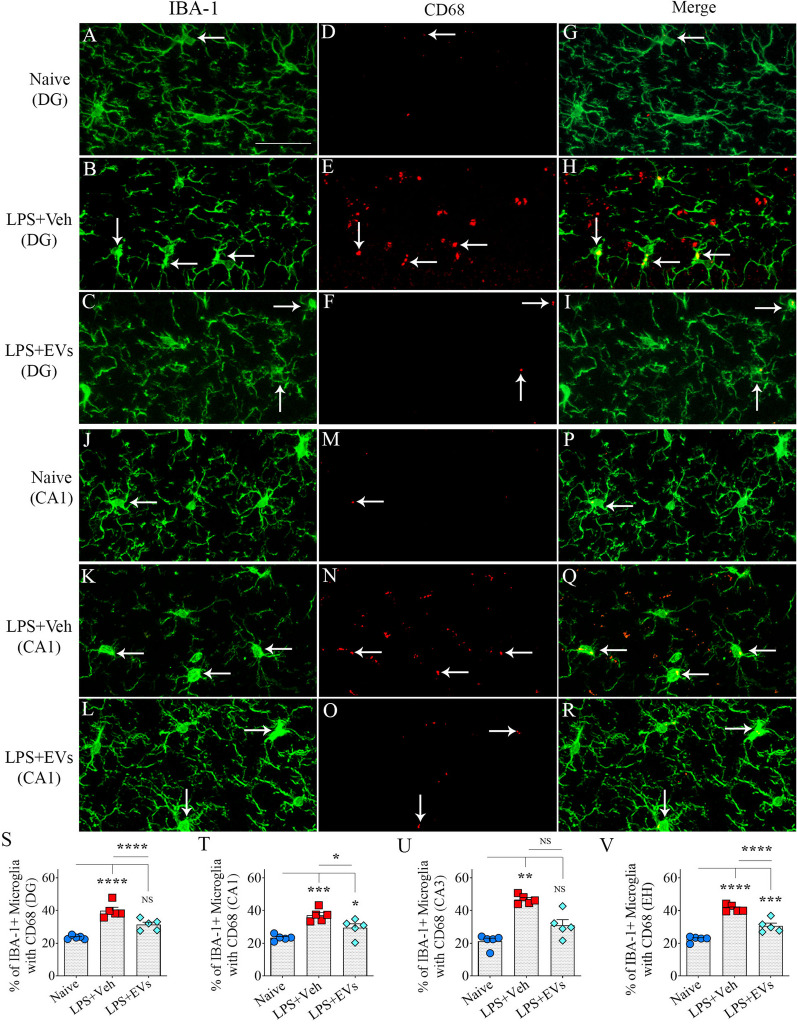
Intranasal administration of extracellular vesicles (EVs) from human induced pluripotent stem cell-derived neural stem cells (hiPSC-NSCs) diminished lipopolysaccharide (LPS)-induced activation of microglia. Images A-R demonstrate examples of IBA-1 + microglia revealing ED-1 + structures (i.e., activated microglia) in the hippocampal dentate gyrus (DG) subfield (**A**-**I**) and the CA1 (**J**-**R**) from naïve control (**A**, **D**, **G** and **J**, **M**, **P**), vehicle-treated LPS (LPS + Veh, **B**, **E**, **H** and **K**, **N**, **Q**), and extracellular vesicle-treated LPS (LPS + EVs, **C**, **F**, **I** and **L**, **O**, **R**) animal groups. The bar charts **S**-**V** compare percentages of IBA-1 + microglia with ED-1 in the dentate gyrus (**S**), the CA1 subfield (**T**), the CA3 subfield (**U**), and the entire hippocampus (EH; **V**) across groups. Scale, **A**–**R** = 25 μm; *, p < 0.05; **, *p* < 0.01; ***, *p* < 0.001, and ****, *p* < 0.0001,; *NS* not significant

Higher percentages of microglia displayed CD68 expression in the vehicle-treated LPS group compared to naïve and EV-treated LPS groups in the dentate gyrus (Fig. 6A–I) and CA1 subfield of the hippocampus (Fig. 6J–R). Compared to naïve mice, the vehicle-treated LPS group exhibited increased percentages of IBA-1 + microglia with CD68 in the DG, CA1, and CA3 subfields of the hippocampus and the entire hippocampus (*p* < 0.01–0.0001. Figure 6S–V). The EV-treated LPS mice also showed increased percentages of such microglia in the CA1 subfield and the entire hippocampus (*p* < 0.05–0.001, Fig. 6S–V), but the increases were significantly less than those seen in vehicle-treated LPS mice (*p* < 0.05–0.0001, Fig. 6S–V). Thus, hiPSC-NSC-EV treatment reduced the activation of microglia.

### hiPSC-NSC-EV treatment inhibited NLRP3 inflammasome activation

**Fig. 7:**
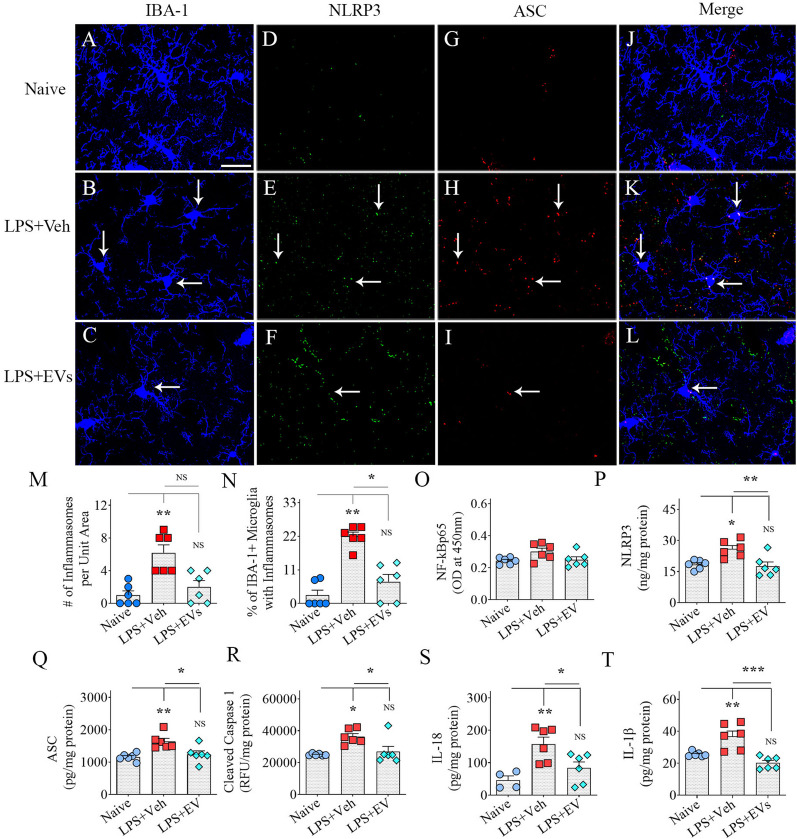
Intranasal administration of extracellular vesicles (EVs) from human induced pluripotent stem cell-derived neural stem cells (hiPSC-NSCs) diminished lipopolysaccharide (LPS)-induced activation of inflammasomes. Images A-L illustrate examples of IBA-1 + microglia displaying NOD-, LRR- and pyrin domain containing 3 (NLRP3) inflammasome complexes (i.e., structures co-expressing NLRP3 and apoptosis-associated speck-like protein containing a caspase recruitment domain [ASC]) in the hippocampal CA3 subfield from naïve control (**A**, **D**, **G**, **J**), vehicle-treated LPS (LPS + Veh, **B**, **E**, **H**, **K**), and extracellular vesicle-treated LPS (LPS + EVs, **C**, **F**, **I**, **L**) animal groups. The bar charts in M-T compare the number of inflammasomes/unit area (M), the percentage of microglia with inflammasomes (**N**), and the concentration of nuclear factor-kappa B p65 subunit (NF-kBp65; **O**), NLRP3 (**P**), ASC (**Q**), cleaved caspase-1 (**R**), interleukin-18 (IL-18; **S**) and IL-1β (**T**) across groups. Scale bar, **A**–**L** = 25 μm; *, *p* < 0.05, **, *p* < 0.01, and ***, *p* < 0.001; *NS* not significant

NLRP3 inflammasome complexes with coexpression of NLRP3 and ASC were observed more frequently in microglia from vehicle-treated LPS group than in naïve and EV-treated LPS groups (Fig. 7A–L). Compared to naive mice, the total numbers of inflammasome complexes per unit area and percentages of microglia displaying inflammasome complexes increased in the vehicle-treated LPS mice (*p* < 0.01) but not in the EV-treated LPS mice (Fig. 7M–N). The percentages of microglia displaying inflammasome complexes were also less in the EV-treated LPS mice compared to the vehicle-treated LPS mice (*p* < 0.05, Fig. 7 [N]). NLRP3 inflammasomes were further evaluated through measurements of the concentration of mediators (NF-kBp65, NLRP3, ASC, and cleaved caspase-1) end products (IL-18 and IL-1β) of NLRP3 inflammasome activation (Fig. 7O–R). The NF-kBp65 showed a trend toward upregulation in the vehicle-treated LPS group compared to the naïve and EV-treated LPS groups, but the differences were statistically insignificant (Fig. 7O). However, compared to naive mice, the concentrations of NLRP3, ASC, cleaved caspase-1, IL-18, and IL-1β were elevated in the vehicle-treated LPS mice (*p* < 0.05–0.01) but not in the EV-treated LPS mice (Fig. 7P–R). The concentrations of all of these proteins were also less in the EV-treated LPS mice compared to the vehicle-treated LPS mice (*p* < 0.05–0.001, Fig. 7S, T). Thus, hiPSC-NSC-EV treatment diminished inflammasome activation within microglia and reduced the secretion of proinflammatory cytokines IL-18 and IL-1β in LPS mice.

### hiPSC-NSC-EV treatment reduced astrocyte hyperplasia and hypertrophy

**Fig. 8:**
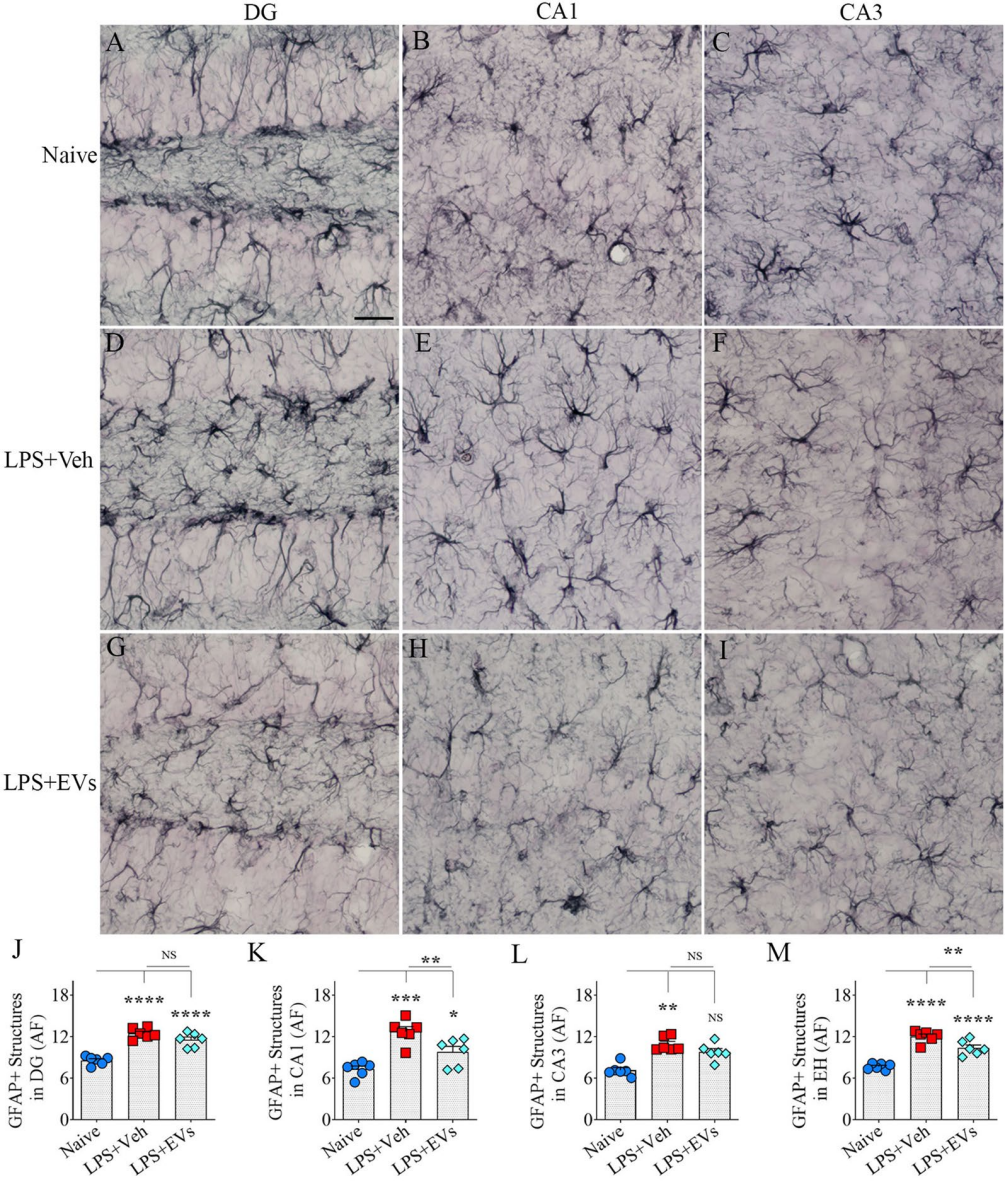
Intranasal administration of extracellular vesicles (EVs) from human induced pluripotent stem cell-derived neural stem cells (hiPSC-NSCs) diminished lipopolysaccharide (LPS)-induced increase in glial fibrillary acidic protein (GFAP +) astrocytic elements. Figures **A**–**I** are examples of GFAP + astrocytic elements in the dentate gyrus (DG; **A**, **D**, **G**), the CA1 subfield (**B**, **E**, **H**), and the CA3 subfield (**C**, **F**, **I**) from naïve control (**A**–**C**), vehicle-treated LPS (LPS + Veh, **D**–**F**), and extracellular vesicle-treated LPS (LPS + EVs, **G**–**I**) animal groups. The bar charts **J**-**M** compare the area fraction (AF) of GFAP + structures in the DG (**J**), the CA1 subfield (**K**), the CA3 subfield (**L**), and the entire hippocampus (EH; **M**) across groups. Scale bar, **A**–**I** = 25 μm; *, *p* < 0.05; **, *p* < 0.01; ***, *p* < 0.001; and ****, *p* < 0.0001; *NS* not significant

The density and morphology of GFAP + astrocytes varied between groups in hippocampal regions. Examples of the distribution of GFAP + astrocytes in the DG and the CA1 and CA3 subfields of the hippocampus from different groups are illustrated (Fig. 8A–I). Compared to naive mice, area fractions of GFAP + structures significantly increased in the DG, the CA1 and CA3 subfields, and the entire hippocampus of the vehicle-treated LPS mice (*p* < 0.01–0.0001, Fig. 8J–M). The EV-treated LPS mice also showed increased area fractions of GFAP + structures in the DG, the CA1 subfield, and the entire hippocampus (p < 0.05–0.0001, Fig. 8J, K, M). However, the increases were significantly less than those seen in vehicle-treated LPS mice in the CA1 subfield and the entire hippocampus (*p* < 0.01, Fig. 8K, M). Thus, hiPSC-NSC-EV treatment reduced the density of GFAP + structures in LPS mice.

### hiPSC-NSC-EV administration to LPS-treated mice prevented hippocampal neurogenesis decline

**Fig. 9:**
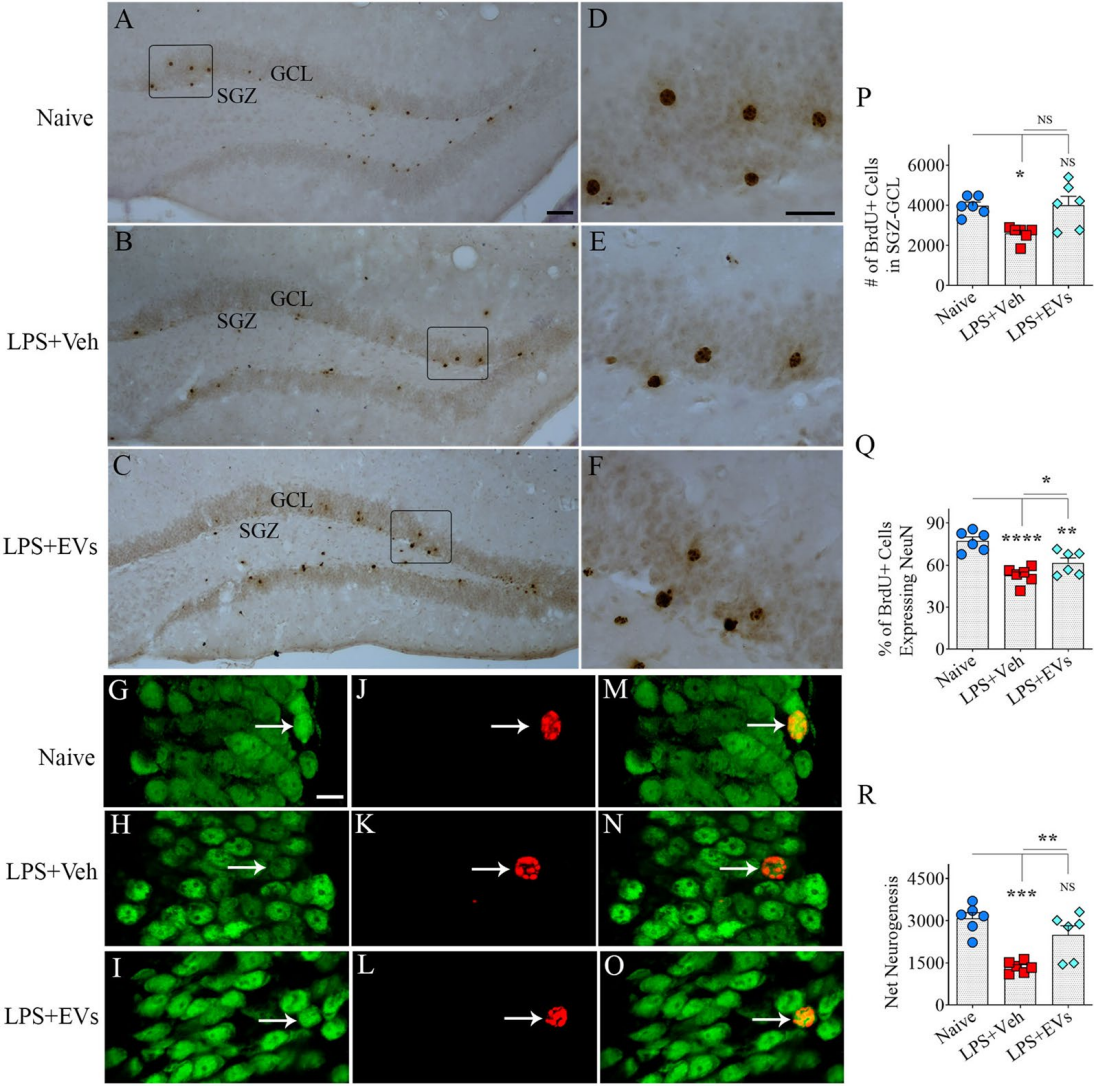
Intranasal administration of extracellular vesicles (EVs) from human induced pluripotent stem cell-derived neural stem cells (hiPSC-NSCs) eased lipopolysaccharide (LPS)-induced hippocampal neurogenesis decline in the third week after LPS injections. Figures **A**–**F** demonstrate examples of newly born cells expressing 5’-bromodeoxyuridine (BrdU) in the subgranular zone-granule cell layer (SGZ-GCL) of the dentate gyrus in the naïve control (**A**, **D**), vehicle-treated LPS (LPS + Veh, **B**, **E**), and extracellular vesicle-treated LPS (LPS + EVs, **C**, **F**). Figures **G**-**O** illustrate the examples of BrdU + newly born cells that have differentiated into mature neurons expressing neuronal nuclear protein (NeuN) in SGZ-GCL of naïve control (**G**, **J**, **M**) LPS + Veh (**H**, **K**, **N**) and LPS + EVs groups (**I**, **L**, **O**). The bar charts **P**-**R** compare the number of BrdU + cells (**P**), the percentage of BrdU + cells expressing NeuN (**Q**), and net neurogenesis (**R**) in SGZ-GCL. Scale bar, **A**–**C** = 50 μm, **D**–**E** = 25 μm; **G**–**O** = 12.5 μm. *, *p* < 0.05; **, *p* < 0.01; ***, *p* < 0.001; and ****, *p* < 0.0001; *NS* not significant

Next, we compared the numbers of new neurons born in the second week after the last LPS injection and survived for seven weeks in LPS mice receiving vehicle or hiPSC-NSC-EVs, in comparison to age-matched naïve control mice through BrdU labeling and BrdU-NeuN dual immunofluorescence methods. Representative images showing the distribution of BrdU + cells in the SGZ-GCL from different groups are illustrated in Fig. 9A–F. One-way ANOVA analysis of the numbers of BrdU + cells in the SGZ-GCL revealed significant differences between different groups of mice (*p* < 0.05, Fig. 9P). The fraction of BrdU + cells in the SGZ-GCL expressing the mature neuronal marker NeuN enabled the quantification of net hippocampus neurogenesis. Figure 9G–O shows representative images of BrdU + cells differentiating into NeuN + neurons in the naive, LPS, and LPS + EV groups. Notably, neuronal differentiation of newborn cells differed significantly across groups (p < 0.0001, Fig. 9Q). The degree of neuronal differentiation was reduced in the LPS + Veh group compared to the naïve control group (*p* < 0.0001, Fig. 9Q). Furthermore, the extent of neuronal differentiation of newly born cells was significantly increased in LPS mice receiving EVs compared to the LPS + Veh group (*p* < 0.05, Fig. 9Q). However, the neuronal differentiation of newly generated cells in the LPS + EV group was less than naïve control group (*p* < 0.01, Fig. 9Q).

Because of the differences in neuronal differentiation, net hippocampal neurogenesis, differed between the groups (*p* < 0.001, Fig. 9R). Net hippocampal neurogenesis was computed by extrapolating the absolute number of BrdU + cells for the entire SGZ-GCL (generated through stereological counts) with the percentage of BrdU + cells that differentiated into NeuN + neurons. When compared to the naïve control group the net hippocampal neurogenesis was reduced in the LPS + Veh group (*p* < 0.001, Fig. 9R). However, the LPS + EVs group displayed comparable net neurogenesis as seen in the naïve control group (*p* > 0.05, Fig. 9R). Furthermore, the LPS + EVs group outscored the LPS + Veh group in the net hippocampal neurogenesis (*p* < 0.01, Fig. 9R). Thus, hIPSC-NSC-EV treatment preserved hippocampal neurogenesis at naïve control levels in the chronic phase of LPS-induced neuroinflammation.

### hiPSC-NSC-EV treatment to LPS-treated mice maintained the status of hippocampal neurogenesis at naive control levels

**Fig. 10:**
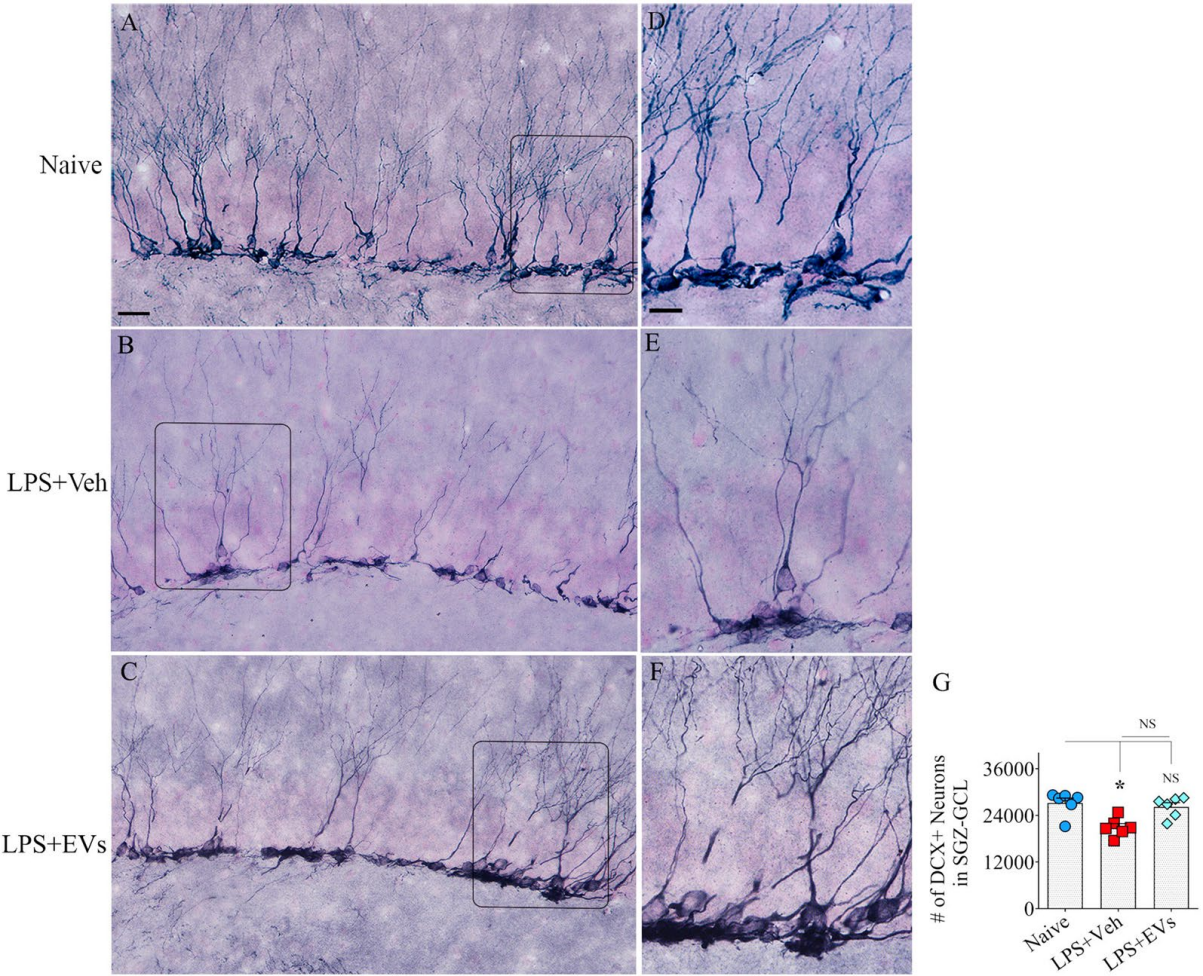
Intranasal administration of extracellular vesicles (EVs) from human induced pluripotent stem cell-derived neural stem cells (hiPSC-NSCs) prevented lipopolysaccharide (LPS)-induced hippocampal neurogenesis decline in the 10th week after LPS injections. **A**–**F** Illustrate examples of doublecortin + (DCX +) newly born neurons in the subgranular zone-granule cell layer (SGZ-GCL) of the dentate gyrus from naïve control (**A**, **D**), vehicle-treated LPS (LPS + Veh, **B**, **E**), and extracellular vesicle-treated LPS (LPS + EVs, **C**, **F**) animal groups. Bar chart G compares the absolute number of DCX + neurons in the SGZ-GCL across groups. Scale bar, **A**–**C** = 50 μm; **D**–**F** = 10 μm. *, *p* < 0.05; *NS* not significant

The density of DCX + newly born neurons varied between groups in the hippocampus. Examples of the distribution of DCX + newly born neurons in the SGZ-GCL from different groups are illustrated (Fig. 10A–F). Compared to naive mice, DCX + newly born neuronal numbers significantly decreased in the vehicle-treated LPS mice (*p* < 0.05) but not in the EV-treated LPS mice (Fig. 10G). Thus, hiPSC-NSC-EV treatment prevented hippocampal neurogenesis decline in LPS mice.

### hiPSC-NSC-EV treatment did not prevent the loss of synaptic proteins

**Fig. 11:**
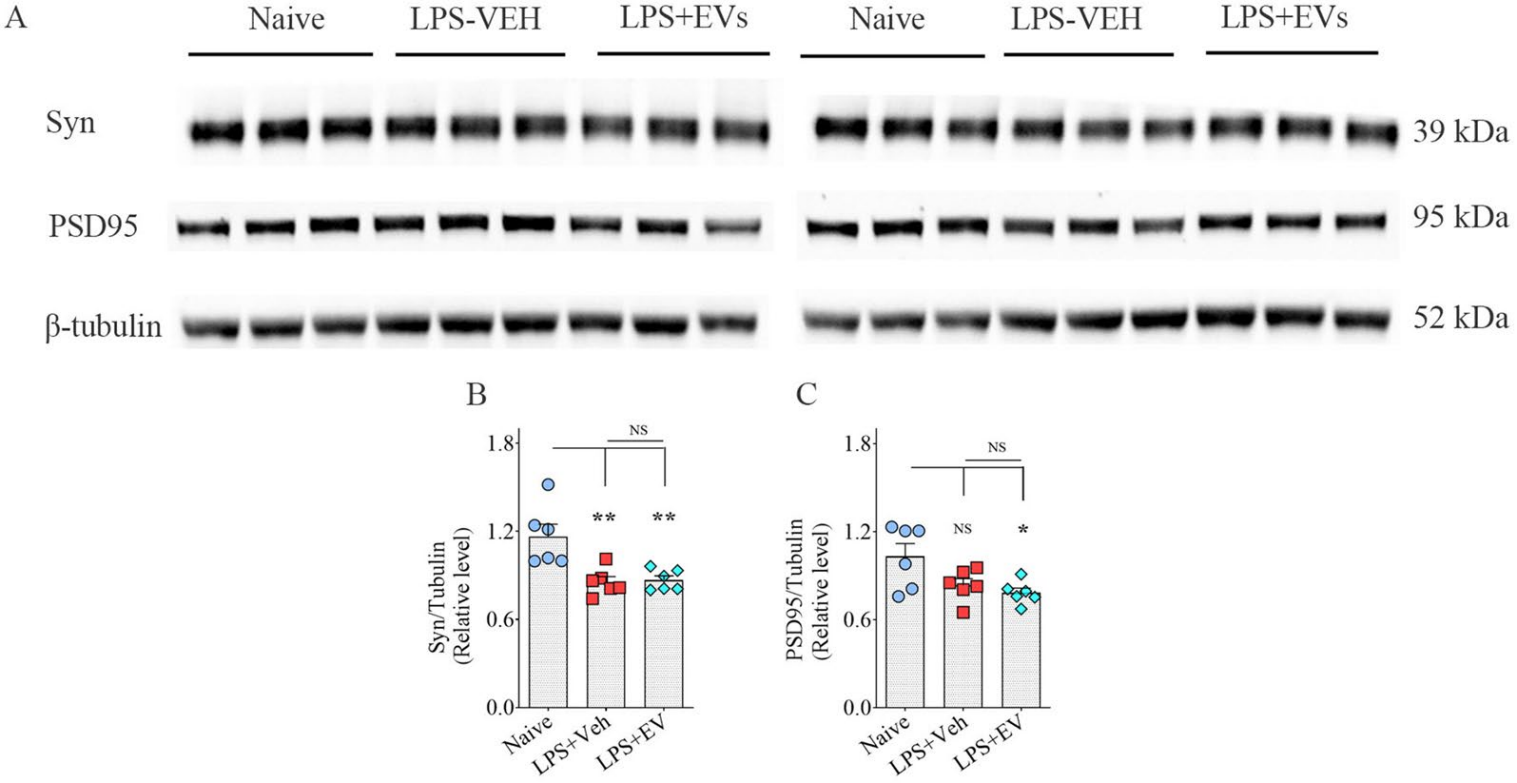
Intranasal administration of extracellular vesicles (EVs) from human induced pluripotent stem cell-derived neural stem cells (hiPSC-NSCs) did not normalize lipopolysaccharide (LPS)-induced reductions in synaptic proteins. **A** Illustrates the western blot bands for synaptophysin (Syn), post-synaptic density protein 95 (PSD95), and β-tubulin proteins from naïve control, vehicle-treated LPS (LPS + Veh), and extracellular vesicle-treated LPS (LPS + EVs). The bar charts **B**–**C** compare the density of Syn (**B**) and PSD 95 (**C**) proteins in the hippocampus. *, *p* < 0.05; **, *p* < 0.01; *NS* not significant

We evaluated Syn + and PSD95 + proteins in the hippocampus of LPS-treated mice receiving the vehicle or hiPSC-NSC-EVs. The LPS + Veh group displayed significantly reduced levels of Syn compared to the naïve control group (*p* < 0.01, Fig. 11A, B). However, the concentration of PSD-95 in the LPS + Veh group did not significantly differ from the naive control group (*p* > 0.05, Fig. 11C). LPS mice receiving hNSC-EVs also exhibited less Syn and PSD95 levels than naive mice (*p* < 0.05–0.01, Fig. 11A–C). Thus, hiPSC-NSC-EV treatment did not prevent reductions in synaptic proteins.

## Discussion

The findings from this study provide novel evidence that IN administrations of hiPSC-NSC-EVs following peripheral inflammation are efficacious for substantially reducing peripheral inflammation-induced chronic neuroinflammation and preventing neuroinflammation-related long-term cognitive impairments. The study also revealed that hiPSC-NSC-EV treatment after LPS-induced neuroinflammation could restrain enduring hippocampal neurogenesis decline.

### Peripheral inflammation caused significant neuroinflammation and adversely affected multiple cognitive processes

Peripheral inflammation, an acute or chronic inflammation occurring outside the central nervous system (CNS), can induce significant inflammatory responses in the brain. Blood-borne factors, including proinflammatory cytokines in the circulating blood could adversely affect the CNS function by interfering with synaptic plasticity and cognitive processes [54]. Such neuroinflammation has been recognized as significant risk factor for developing dementia, including AD [55]. While the role of peripheral inflammation as the cause or the effect of AD is still debated, studies have reported that AD is associated with elevated levels of multiple proinflammatory proteins in the blood [56] and cognitively normal individuals exhibiting increased concentrations of proinflammatory markers in blood have a higher risk for future mild cognitive impairment (MCI), AD dementia, and all-cause dementia [57–60]. Also, patients surviving sepsis develop chronic neuroinflammation and acute and mental disorders [61]. Moreover, individuals maintaining higher levels of inflammatory proteins over time exhibit worse brain-related outcomes [62, 63]. Studies from animal models also support peripheral inflammation impacting brain function. For example, chronic low-grade systemic inflammation caused by Polyl:C in a mouse model can induce peripheral and central inflammation associated with memory impairments, tauopathy, activated microglia, and altered gene expression [64]. Similarly, chronic periodontitis can lead to cognitive decline [65]. A study has implied that systemic alarmins and proinflammatory cytokines such as IL-1β, TNFα, and high-mobility group box 1 proteins can trigger neuroinflammation after peripheral inflammation [66].

LPS administration at low doses can also recapitulate the effects of mild infection and peripheral inflammation, which can exacerbate AD pathogenesis [67] Accordingly, the LPS model employed in the current study induced systemic inflammation and neuroinflammation immediately following low-dose, daily intraperitoneal administrations of LPS for 7 days. Chronic neuroinflammation in this model was characterized by the activation of microglia presenting increased concentration of CD68 protein, NLRP3 inflammasome complexes, and elevated levels of various NLRP3 inflammasome mediators (NLRP3, ASC, and cleaved caspase-1) and end products (IL-1β and IL-18) in the hippocampus. Such chronic neuroinflammatory milieu in the hippocampus also reduced neurogenesis. Notably, the above inflammatory processes in the brain and hippocampal neurogenesis decline adversely affected the cognitive processes for encoding associative recognition memory [38]. Furthermore, hippocampus-specific cognitive functions were considerably impaired. These include diminished proficiency for recognizing and generating temporal patterns by encoding patterns over time in a temporal pattern processing task requiring the integrity of the hippocampal CA1 subfield [39, 40] and a reduced competence for distinguishing similar but not identical experiences by encoding similar representations in a non-overlapping manner in a pattern separation task [42, 43]. However, LPS treatment only mildly altered social interaction behavior [45, 68]. Thus, the animal model of peripheral inflammation employed in the study induced significant chronic neuroinflammation and impacted cognitive processes, supporting the perceived crosstalk between peripheral inflammation and neuroinflammation [69].

### hiPSC-NSC-EV treatment after LPS-induced peripheral inflammation eased cognitive impairments by restraining neuroinflammation

In this study, we chose to test the proficiency of hiPSC-NSC-EVs in curtailing neuroinflammatory cascades induced by peripheral inflammation because our earlier studies have implied that these EVs exhibit robust antiinflammatory properties, evident from multiple assays [31]. First, these EVs, expressing multiple EV-specific markers such as CD63, CD81, and ALIX and lacking deep cellular proteins, displayed a robust ability to suppress the release of IL-6 from LPS-stimulated mouse macrophages in vitro in a dose-dependent manner and to significantly dampen the cytokine storm in the hippocampus following an episode of status epilepticus [31]. Second, hiPSC-NSC-EVs significantly restrained the release of IL-1β and TNFα from LPS-stimulated human microglia derived from hiPSCs [32]. Third, small RNA sequencing and proteomic studies revealed that these EVs are naturally enriched with multiple antiinflammatory miRNAs and proteins [31], of which miR-21-5p and pentraxin-3 (PTX3) stand out because the knockdown or inhibition of miR-21-5p and PTX3 significantly reduced the antiinflammatory effects of hiPSC-NSC-EVs on LPS-stimulated human microglia in our recent study [32]. Antiinflammatory effects of miR-21-5p are linked to its ability to regulate NF-kB signaling, increase the concentration of IL-10, and inhibit the release of TNFα [70–73]. PTX3 can protect the blood–brain barrier, activate the beneficial type 2 astrocytes, and regulate neutrophil migration into the brain in inflammatory conditions [74–76]. hiPSC-NSC-EVs are also enriched with miR-103a, capable of reducing neuroinflammation by inhibiting prostaglandin-endoperoxide synthase 2 [31, 77], hemopexin proficient in influencing the transformation of proinflammatory microglia into antiinflammatory microglia [78], and galectin-3 binding protein adept in reducing NF-kB signaling pathway [79]. Our results demonstrated that IN administrations of hiPSC-NSC-EVs following seven days of LPS treatment can prevent a multitude of cognitive impairments linked to neuroinflammation in several brain regions following LPS-induced peripheral inflammation. These were evident from improved recognition memory function and better performance in temporal pattern processing, and pattern separation tasks in LPS-treated mice receiving hiPSC-NSC-EVs, compared to LPS-treated mice receiving the vehicle. Importantly, improved cognitive functions in LPS-treated mice receiving hiPSC-NSC-EVs were associated with considerable suppression of neuroinflammation compared to LPS-treated mice receiving the vehicle. Such effects in LPS-treated mice receiving hiPSC-NSC-EVs were evidenced by diminished microgliosis, reduced percentages of microglia presenting CD68, and NLPR3 inflammasome complexes. Furthermore, hiPSC-NSC-EV treatment diminished the concentration of mediators (NLPR3, ASC, and cleaved caspase-1) and end products (IL-1β and IL-18) of NLRP3 inflammasome activation in the hippocampus. Such changes reflect substantial antiinflammatory effects as NLRP3 inflammasome activation is one of the mechanisms by which activated microglia contribute to a chronic neuroinflammatory state in the brain through downstream signaling of IL-18 and IL-1β resulting in hyperactivation of p38/MAPK signaling and continuous release of multiple proinflammatory cytokines [35, 80].

Consistent with the above changes, LPS-treated mice receiving hiPSC-NSC-EVs displayed a morphology of microglia suggestive of the transformation of proinflammatory phenotypes into noninflammatory phenotypes with an extensive ramification of processes [49]. Such positive modulation of microglial morphology and function in inflammatory conditions has considerable significance because reactive microglia play a major role in the pathogenesis of neurodegenerative diseases [81–84] and sustained microglial activation can promote neuronal dysfunction and neurodegeneration [85]. It is plausible that the cargos released from hiPSC-NSC-EVs internalized by microglia promote the transformation of microglia into noninflammatory phenotypes through transcriptomic changes. Such direct effects are likely because our recent study in a 5 × familial Alzheimer’s disease (5xFAD) mouse model has demonstrated that hiPSC-NSC-EVs directly target microglia, including reactive and plaque-associated microglia, within 45 min after an IN administration [86]. However, additional studies are needed to determine the extent of gene expression changes in activated microglia after incorporating IN-administered hiPSC-NSC-EVs. Besides, astrocyte hypertrophy was reduced in the hippocampus of LPS-treated mice receiving hiPSC-NSC-EVs implying that the antiinflammatory effects of these EVs extended to astrocytes too. Thus, improvements in multiple cognitive processes in LPS-treated mice receiving hiPSC-NSC-EVs observed in this study are linked primarily to substantial suppression of neuroinflammation following peripheral inflammation. Such interpretation is also supported by studies implying that neuroinflammation alone can impact cognitive function, as neuroinflammation can promote progressive synaptic and neuronal loss and pathological changes in neural network function [87, 88]. However, the influence of other proteins in the cargo of hiPSC-NSC-EVs cannot be ruled out, as these EVs are also enriched with proteins such as agrin capable of facilitating long-term potentiation and hippocampal neurogenesis [89, 90].

Moreover, the efficacy of hiPSC-NSC-EVs to curtail neuroinflammation after LPS-induced peripheral inflammation, as observed in this study, has considerable relevance for developing an effective biologic for preventing or reducing the incidence of AD because neuroinflammatory conditions develop much before the onset of clinical symptoms in AD [4]. Neuroinflammation after brain infections could also lead to AD [25, 26]. Thus, IN administration of hiPSC-NSC-EVs could be employed in individuals displaying chronic peripheral inflammation related neuroinflammation to prevent them from developing MCI, AD dementia, or dementia of any type. Individuals with significant chronic peripheral inflammation could be easily diagnosed through longitudinal analysis of proinflammatory cytokines in the plasma, whereas individuals with chronic neuroinflammation can be parsed out through periodic characterization of brain-derived extracellular vesicles in the plasma [39, 91, 92].

### hiPSC-NSC-EV treatment prevented hippocampal neurogenesis decline but not synapse loss following LPS-induced peripheral inflammation and neuroinflammation

Neuroinflammation resulting from peripheral inflammation can also impact hippocampal neurogenesis, as neuroinflammatory conditions create an unfavorable milieu for adult neurogenesis [93, 94]. Activated microglia releasing increased concentrations of proinflammatory cytokines can diminish neurogenesis by inhibiting NSC proliferation and the recruitment of new neurons into hippocampal networks involved in cognitive and memory processes [95–99]. On the other hand, antiinflammatory microglia secreting IL-4 and IL-10 and transforming growth factor-beta can boost neurogenesis by increasing NSC proliferation and the neuronal differentiation of newly born cells and supporting the long-term survival of newly differentiated neurons [96, 97, 100, 101]. In the current study, net hippocampal neurogenesis was substantially reduced in LPS-treated mice receiving the vehicle. However, LPS-treated mice receiving hiPSC-NSC-EVs maintained a similar extent of neurogenesis as age-matched naïve control mice. Thus, normalized neurogenesis in LPS-treated mice receiving hiPSC-NSC-EVs is likely a downstream beneficial effect of EVs suppressing neuroinflammation. However, involvement of pro-neurogenic pathways, such as the activation of brain-derived neurotrophic factor-extracellular signal-regulated kinase-cyclic AMP response-element binding protein signaling, as observed in a traumatic brain injury model following the administration of human bone marrow mesenchymal stem cell-derived EVs, cannot be ruled out [48]. Such possibility is supported by hiPSC-NSC-EVs exhibiting enriched payload of proteins such as agrin efficient in promoting CREB activation [89] and agrin and PTX3 capable of directly enhancing neurogenesis [90, 102]. LPS-treated mice receiving the vehicle also exhibited significantly reduced levels of synaptic proteins, such as presynaptic protein Syn and the post-synaptic protein PSD95 in the hippocampus. However, hiPSC-NSC-EV treatment did not normalize the concentration of synaptic proteins to age-matched naïve control levels, implying that synaptic recovery did not contribute to better cognitive function in LPS-treated mice receiving hiPSC-NSC-EVs.

## Conclusions

IN administrations of hiPSC-NSC-EVs in conditions causing chronic neuroinflammation can substantially modulate microglial activation and prevent hippocampal neurogenesis decline. Notably, such robust antiinflammatory and pro-neurogenic effects of hiPSC-NSC-EVs were adequate for maintaining proficiency for multiple cognitive processes. Thus, IN administrations of hiPSC-NSC-EVs are likely valuable in neurological and neurodegenerative conditions exhibiting chronic neuroinflammation for controlling neuroinflammation and preventing sustained cognitive dysfunction. Since this study was performed only in male mice, future studies in disease models need to confirm whether similar effects could be obtained in both sexes. Furthermore, specific effects of hiPSC-NSC-EVs need to be validated using EVs from cell types lacking therapeutic effects (e.g., fibroblast-derived EVs).

### Supplementary Information

**Additional file 1: Figure S1.** *Human induced pluripotent stem cells derived neural stem cells (hIPSC-NSCs) express specific markers. *Images A-D illustrate that all cells in the passage 11 NSCs derived from hiPSCs express NSC markers Nestin (A) and Sox-2 (B). Scale bar, 100 μm. **Figure S2.** *Seven days of Lipopolysaccharide (LPS) administration induced systemic inflammation and neuroinflammation when examined a day after the last LPS injection.* The bar charts A-J compare the concentrations of proinflammatory cytokines in the liver (A-D), serum (E-H), and hippocampus (I-L), such as tumor necrosis factor-alpha (TNF-α, A, E, I); interleukin beta (IL-1β, B, F, J); IL-6 (DC, G, K); and IL-18 (D, H, L). *, p < 0.05; **, p < 0.01; ***, p < 0.001; NS, not significant. **Figure S3.** *Intranasally administered hiPSC-NSC-EVs incorporated into NeuN + neurons and in multiple brain regions of lipopolysaccharide (LPS) treated mice.* A-L: Images illustrating the incorporation of EVs into NeuN+ neurons in the medial prefrontal cortex (mPFC; A, B), the somatosensory cortex (SSC; C, D), dentate granule cell layer (GCL; E, F), the CA1 cell layer (G, H), the CA3 cell layer (I, J) and midbrain (MB; K, L) in LPS-treated mice 6 hours post-administration. The images in B, D, F, H, J, and L represent the magnified versions of images A, C, E, G, I, and K indicated in boxes. Scale bar-A-L, 12.5 μm. **Figure S4.** *Intranasally administered hiPSC-NSC-EVs incorporated into IBA-1+ microglia in multiple brain regions of lipopolysaccharide (LPS) treated mice.* A-L: Images illustrating the incorporation of EVs into IBA-1+ microglia in the medial prefrontal cortex (mPFC; A, B), the somatosensory cortex (SSC; C, D), dentate granule cell layer (GCL; E, F), the CA1 cell layer (G, H), the CA3 cell layer (I, J) and midbrain (MB; K, L) in LPS-treated mice 6 hours post-administration. The images in B, D, F, H, J, and L represent the magnified versions of images A, C, E, G, I, and K indicated in boxes. Scale bar-A-L, 12.5 μm. **Figure S5.** *Intranasally administered hiPSC-NSC-EVs incorporated into GFAP+ astrocytes in the hippocampus of lipopolysaccharide (LPS)-treated mice.* The figure illustrates the incorporation of EVs into GFAP + astrocytes in the CA1 (A) and CA3 (B) subfields of the hippocampus of Lipopolysaccharide (LPS) treated mice at 6 hours post-administration—scale bar, 12.5 μm. **Figure S6.** Additional data of the object-in-place test (OIPT), temporal pattern processing task (TPPT), and pattern separation test (PST). The bar charts A-B compare the total object exploration times (TOETs) in T2 and T3 across groups in OIPT. The bar charts M-P compare the TOETs in T2–T5 across groups in TPPT. The bar charts G-H compare the TOETs in T2 and T3. The bar charts in G-I compare the TOETs in T2–T4 across groups in PST. *, p < 0.05; **, p < 0.01; NS, not significant. **Figure S7.** (Blots with corresponding inverts for EV markers shown in Fig. 1). **Figure S8.** (Blots with corresponding original membrane for EV markers shown in Fig. 1). **Figure S9.** (Blots with corresponding inverts for synaptophysin and PSD95 shown in Fig. 11). **Figure S10.** (Blots with corresponding original membrane for synaptophysin and PSD95 shown in Fig. 11.

## Data Availability

Data supporting this study are included within the article.
