# Supplementary material for: Extracellular vesicles from hiPSC-NSCs can prevent peripheral inflammation-induced cognitive dysfunction with inflammasome inhibition and improved neurogenesis in the hippocampus
Source: J Neuroinflammation. 2023 Dec 12;20:297. doi: 10.1186/s12974-023-02971-y (PMC10717852; doi:10.1186/s12974-023-02971-y)

**Additional Document**

**Contents**

1. Supplemental Figure 1
2. Supplemental Figure 2
3. Supplemental Figure 3
4. Supplemental Figure 4
5. Supplemental Figure 5
6. Supplemental Figure 6
7. Supplemental Figure 7
8. Supplemental Figure 8
9. Supplemental Figure 9
10. Supplemental Figure 10
11. **Figure S1**

**
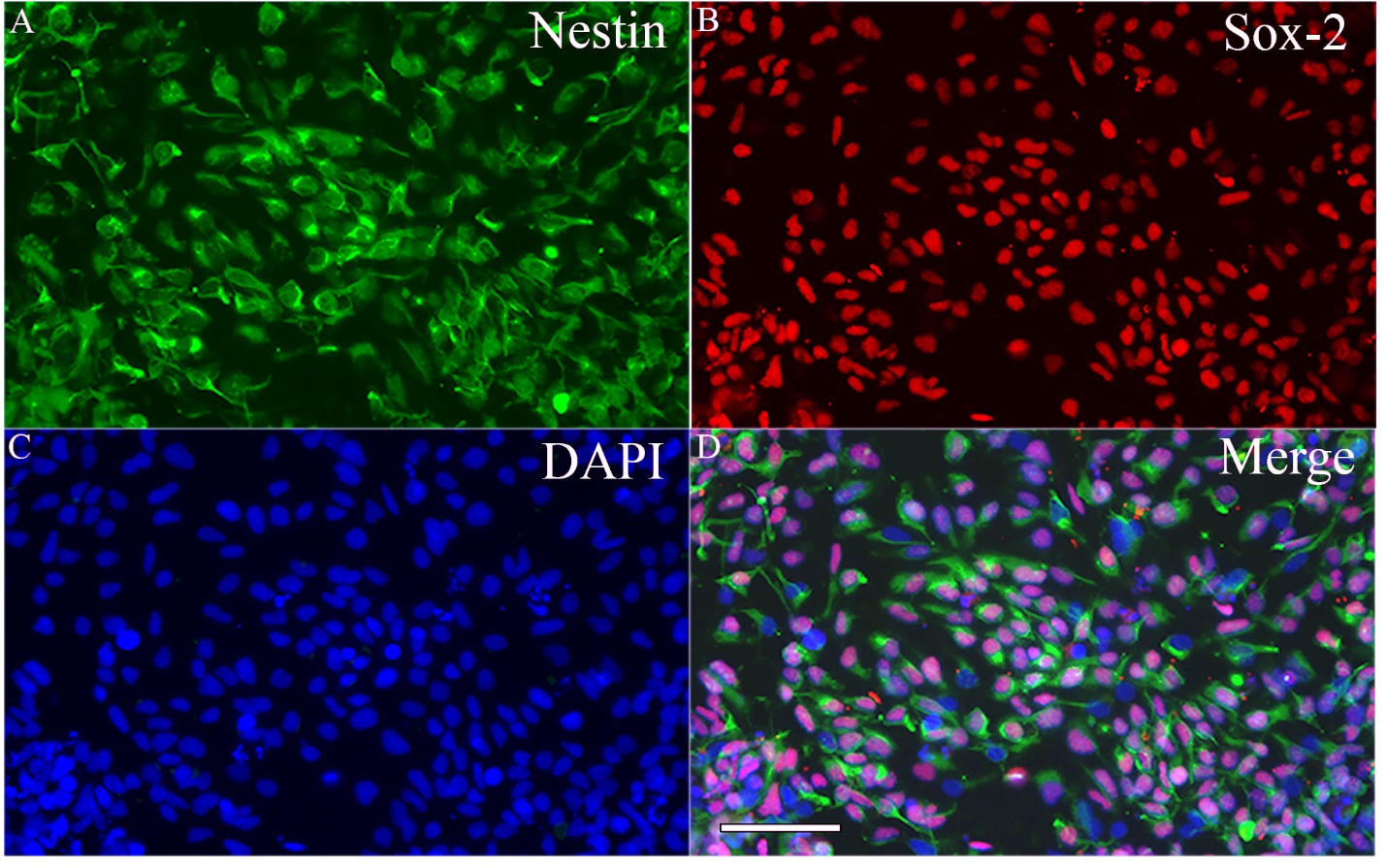
**

**Figure S1 –** *Human induced pluripotent stem cells derived neural stem cells (hIPSC-NSCs) express specific markers.* Images A-D illustrate that all cells in the passage 11 NSCs derived from hiPSCs express NSC markers Nestin (A) and Sox-2 (B). Scale bar, 100 μm.

**Figure S2**

**
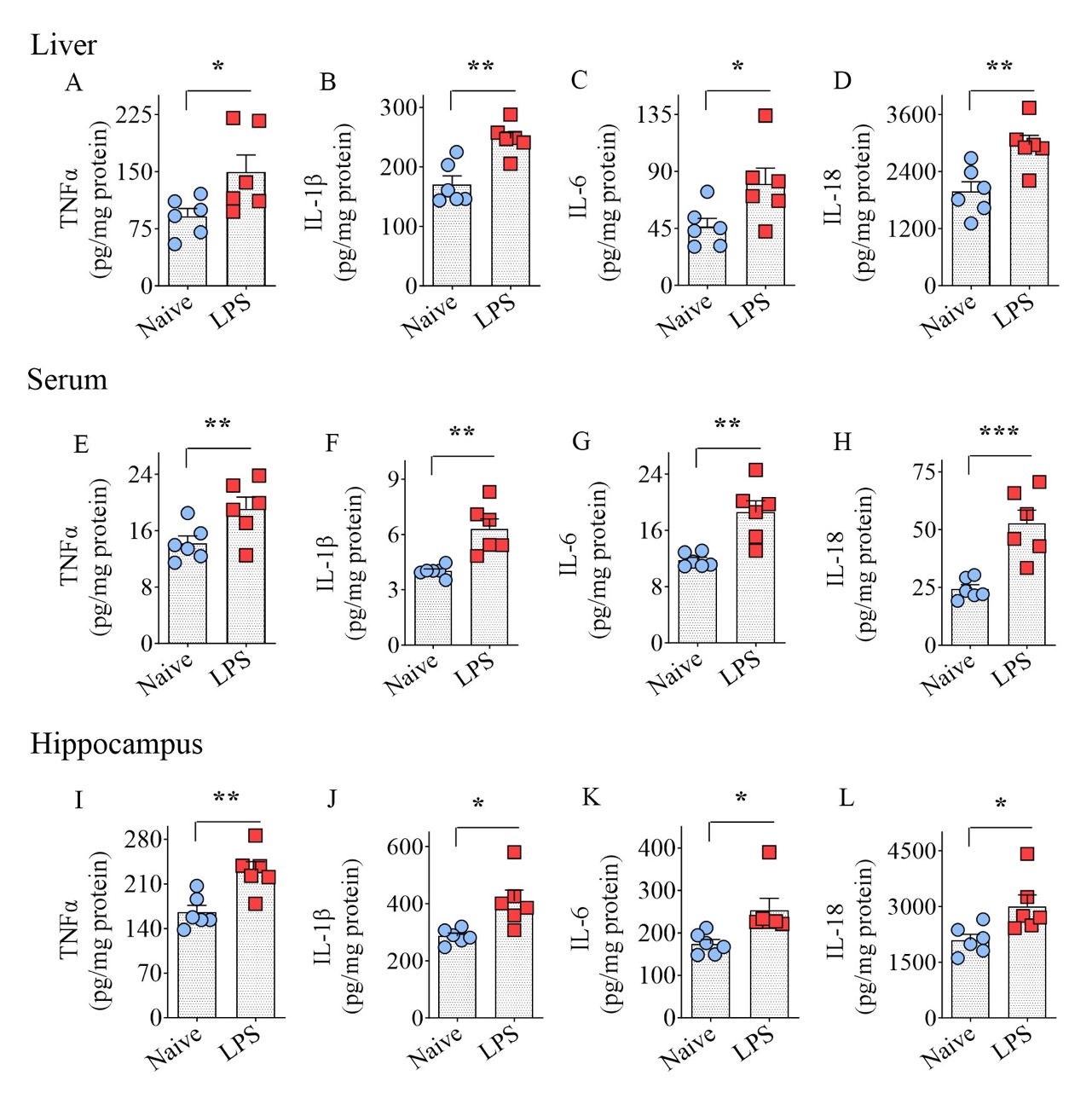
**

**Figure S2 -** *Seven days of Lipopolysaccharide (LPS) administration induced systemic inflammation and neuroinflammation when examined a day after the last LPS injection.* The bar charts A-J compare the concentrations of proinflammatory cytokines in the liver (A-D), serum (E-H), and hippocampus (I-L), such as tumor necrosis factor-alpha (TNF-α, A, E, I); interleukin beta (IL-1β, B, F, J); IL-6 (DC, G, K); and IL-18 (D, H, L). *, p < 0.05; **, p < 0.01; ***, p < 0.001; NS, not significant.

**Figure S3**

**
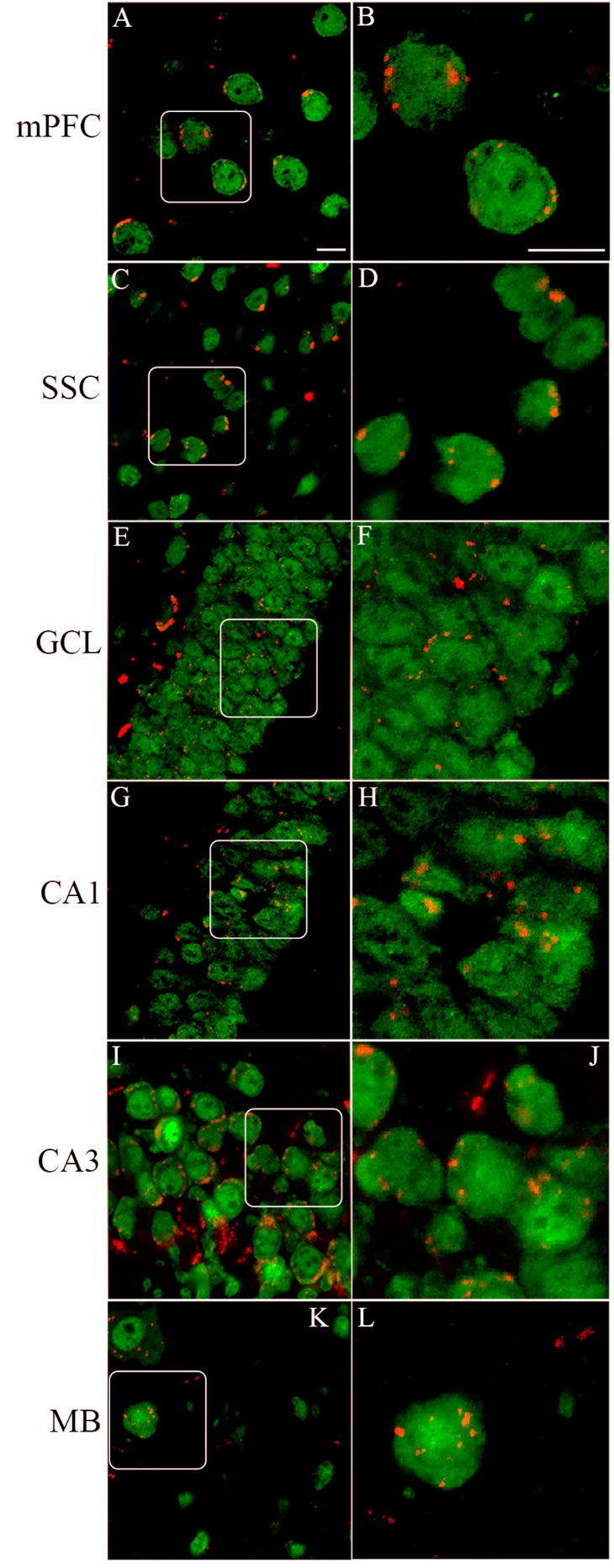
**

**Figure S3.** *Intranasally administered hiPSC-NSC-EVs incorporated into NeuN + neurons and in multiple brain regions of lipopolysaccharide (LPS) treated mice.* A-L: Images illustrating the incorporation of EVs into NeuN+ neurons in the medial prefrontal cortex (mPFC; A, B), the somatosensory cortex (SSC; C, D), dentate granule cell layer (GCL; E, F), the CA1 cell layer (G, H), the CA3 cell layer (I, J) and midbrain (MB; K, L) in LPS-treated mice 6 hours post-administration. The images in B, D, F, H, J, and L represent the magnified versions of images A, C, E, G, I, and K indicated in boxes. Scale bar-A-L, 12.5 μm.

**Figure S4**


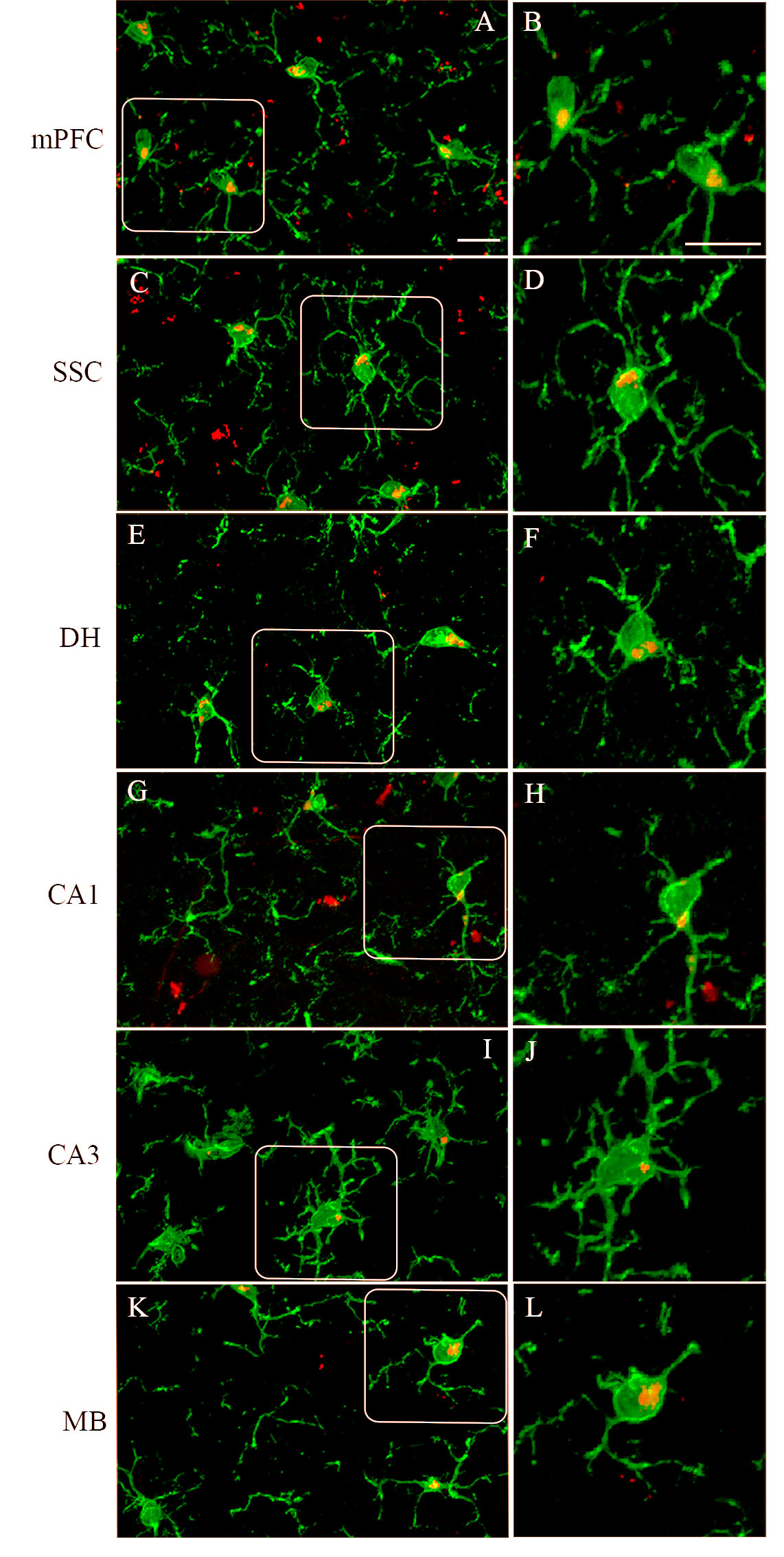


**Figure S4.** *Intranasally administered hiPSC-NSC-EVs incorporated into IBA-1+ microglia in multiple brain regions of lipopolysaccharide (LPS) treated mice.* A-L: Images illustrating the incorporation of EVs into IBA-1+ microglia in the medial prefrontal cortex (mPFC; A, B), the somatosensory cortex (SSC; C, D), dentate granule cell layer (GCL; E, F), the CA1 cell layer (G, H), the CA3 cell layer (I, J) and midbrain (MB; K, L) in LPS-treated mice 6 hours post-administration. The images in B, D, F, H, J, and L represent the magnified versions of images A, C, E, G, I, and K indicated in boxes. Scale bar-A-L, 12.5 μm.

**Figure S5**

**
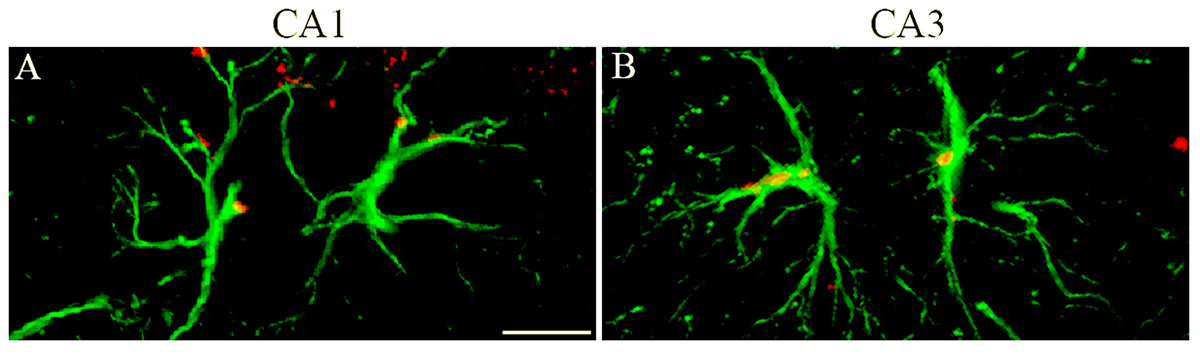
**

**Figure S5.** *Intranasally administered hiPSC-NSC-EVs incorporated into GFAP+ astrocytes in the hippocampus of lipopolysaccharide (LPS)-treated mice.* The figure illustrates the incorporation of EVs into GFAP + astrocytes in the CA1 (A) and CA3 (B) subfields of the hippocampus of Lipopolysaccharide (LPS) treated mice at 6 hours post-administration—scale bar, 12.5 μm.

**Figure S6**

**
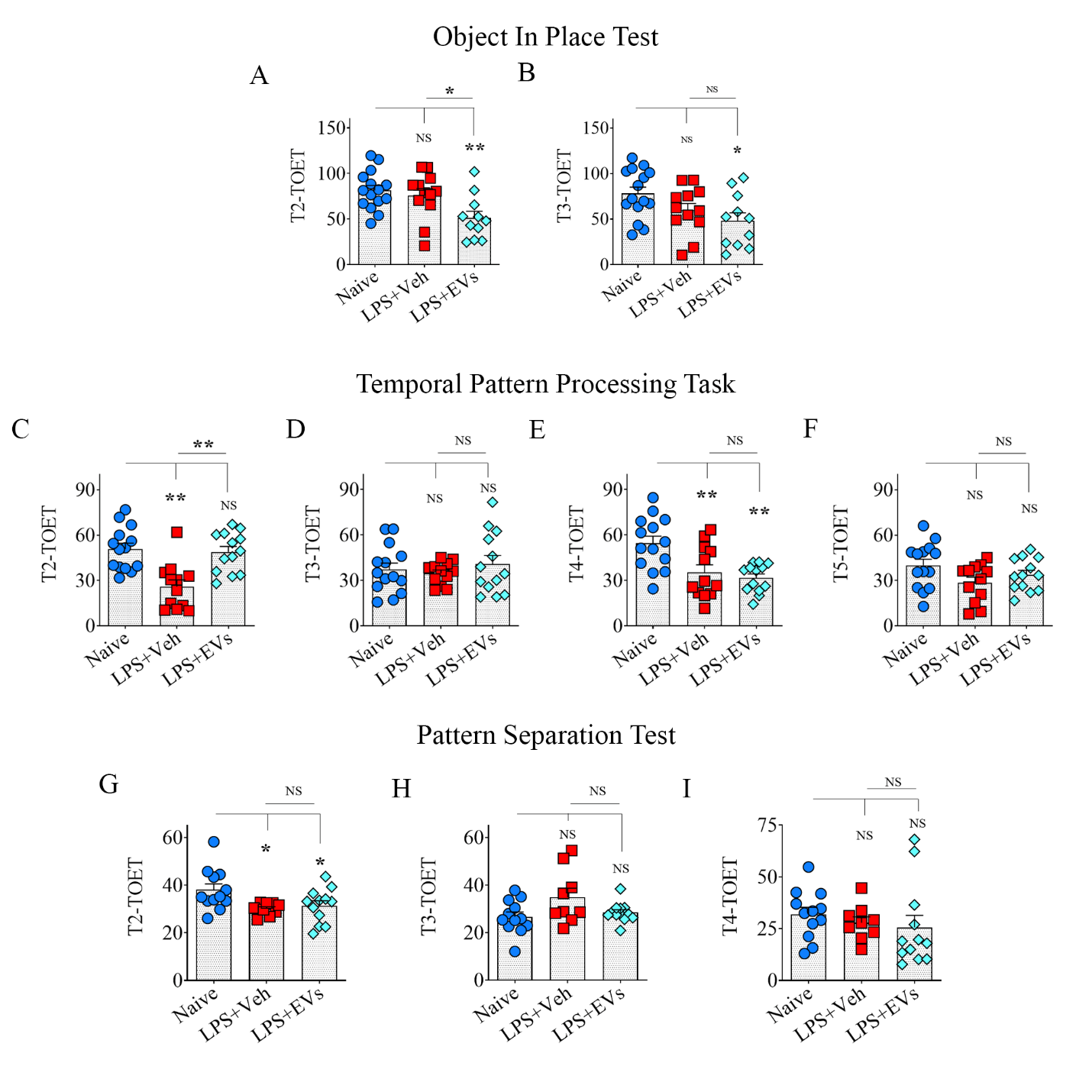
**

**Figure S6. Additional data of the object-in-place test (OIPT), temporal pattern processing task (TPPT), and pattern separation test (PST). The bar charts A-B compare the total object exploration times (TOETs) in T2 and T3 across groups in OIPT. The bar charts M-P compare the TOETs in T2-T5 across groups in TPPT. The bar charts G-H compare the TOETs in T2 and T3. The bar charts in G-I compare the TOETs in T2-T4 across groups in PST. *, p < 0.05; **, p < 0.01; NS, not significant.**

**Figure S7**


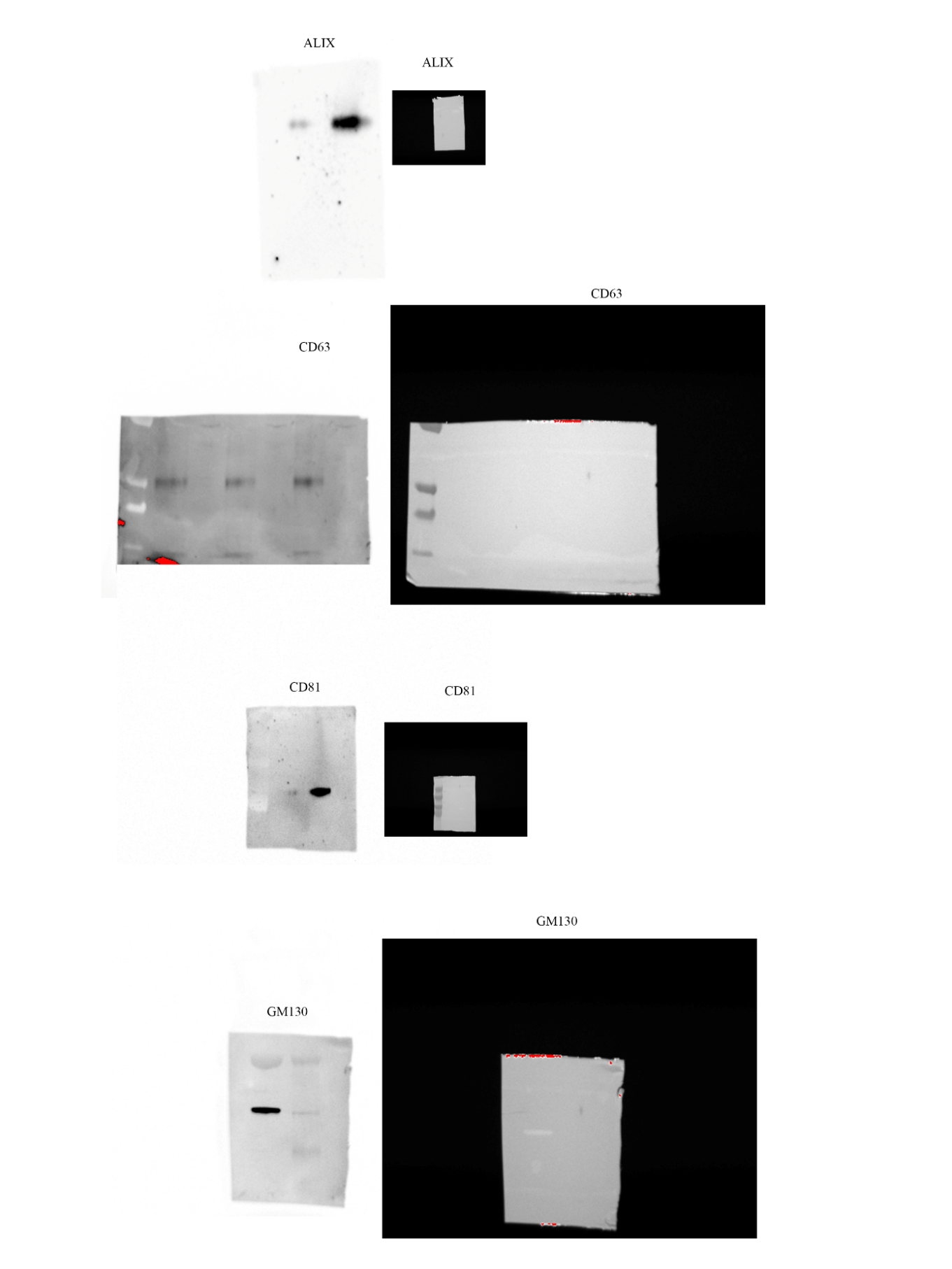
(**Blots with corresponding inverts for EV markers shown in figure 1)**

**Figure S8**

**
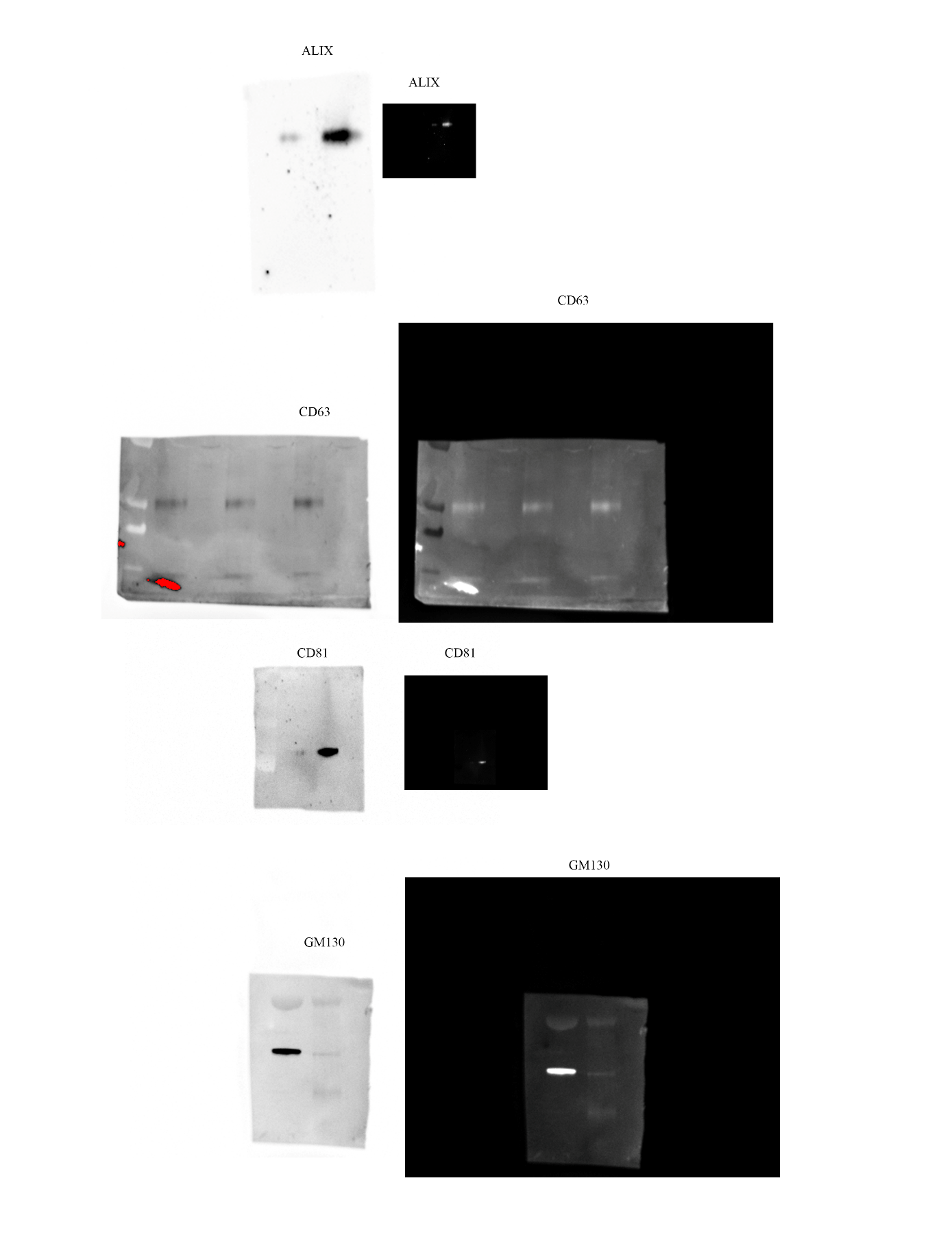
**(**Blots with corresponding original membrane for EV markers shown in figure 1)**

**Figure S9**

(**Blots with corresponding inverts for synaptophysin and PSD95 shown in figure 11)**

**
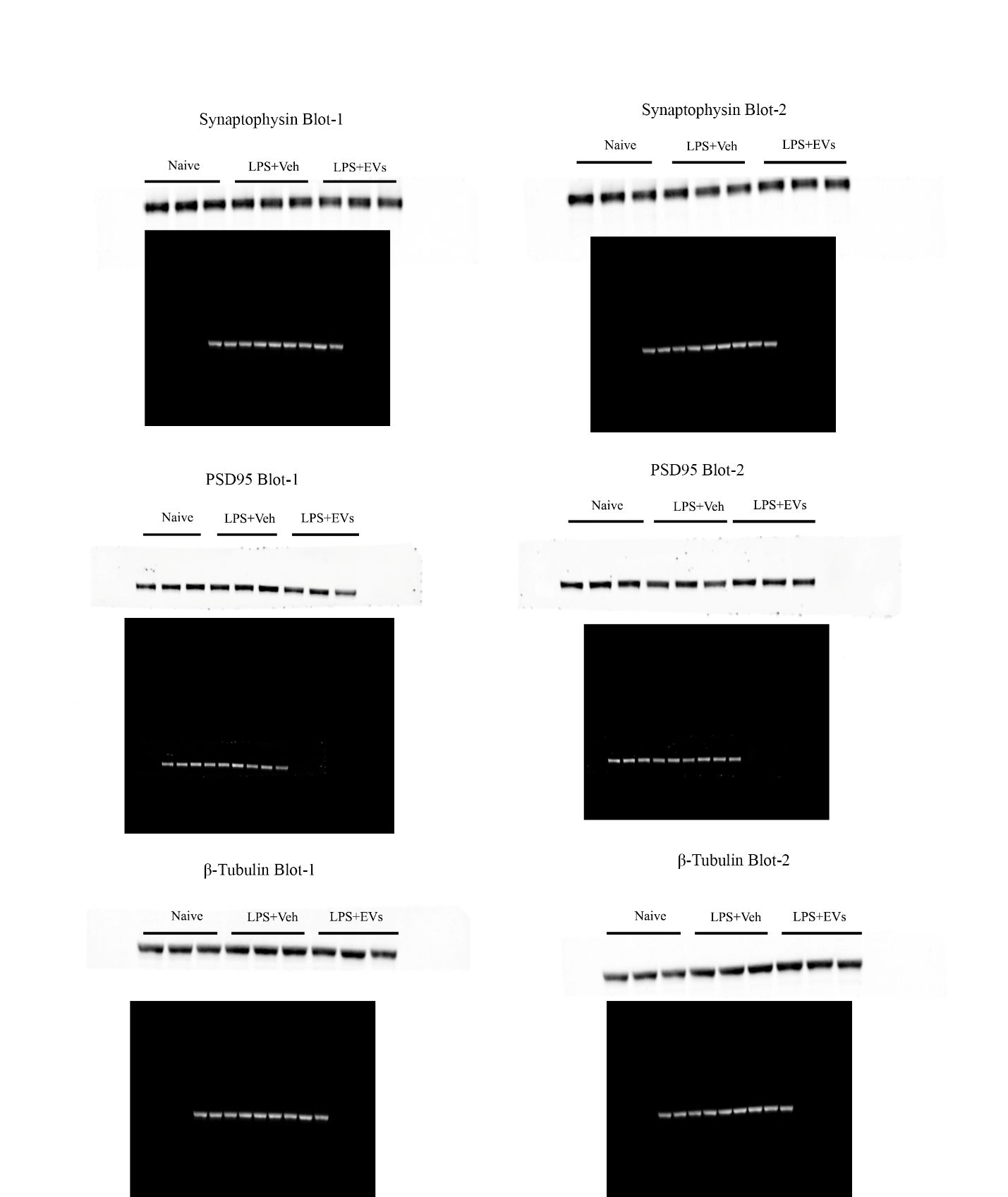
**

**Figure S10**

(**Blots with corresponding original membrane for synaptophysin and PSD95 shown in figure 11)**


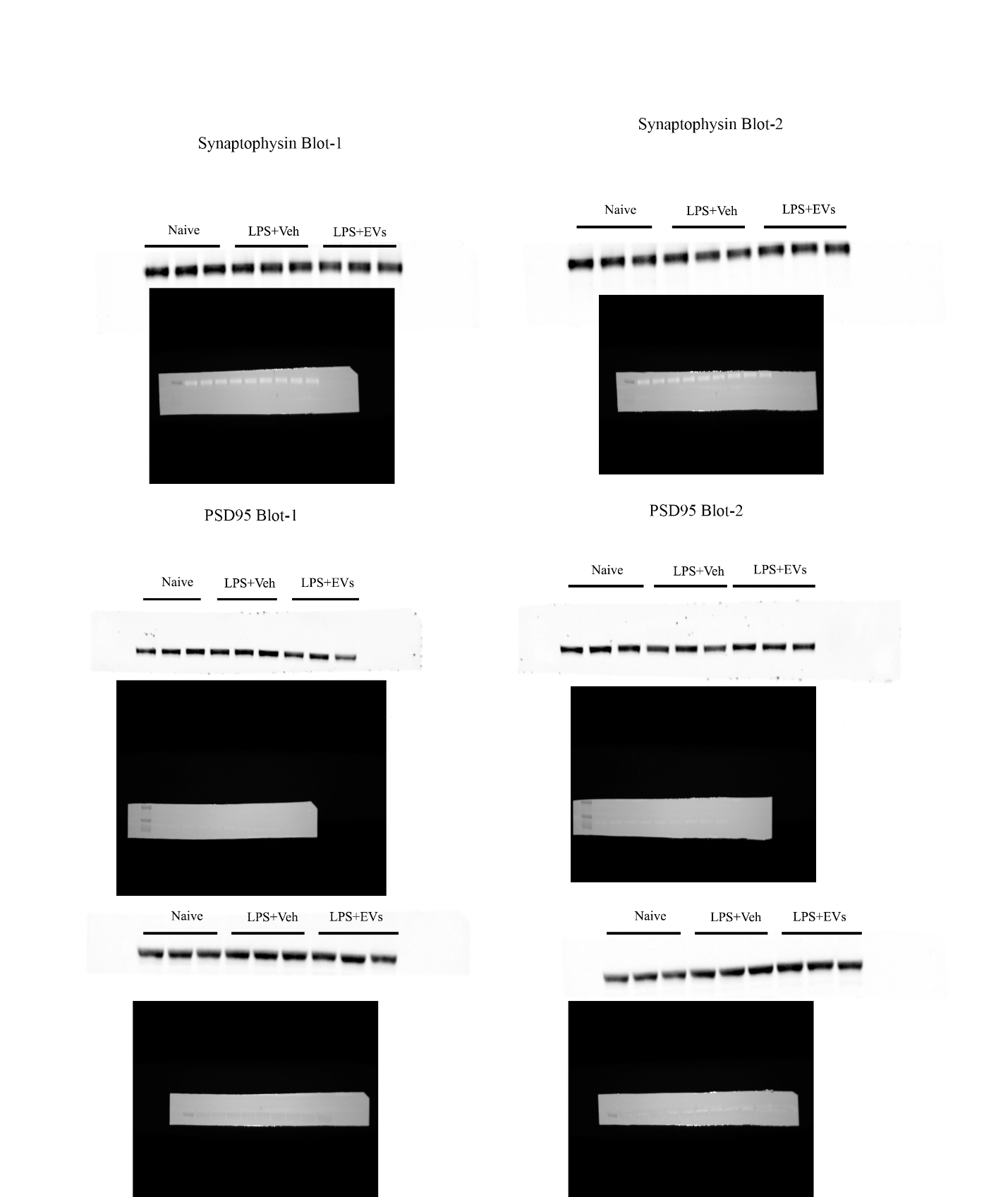

Supplement: Supplementary file 1 — Additional file 1: Figure S1. Human induced pluripotent stem cells derived neural stem cells (hIPSC-NSCs) express specific markers. Images A-D illustrate that all cells in the passage 11 NSCs derived from hiPSCs express NSC markers Nestin (A) and Sox-2 (B). Scale bar, 100 μm. Figure S2. Seven days of Lipopolysaccharide (LPS) administration induced systemic inflammation and neuroinflammation when examined a day after the last LPS injection. The bar charts A-J compare the concentrations of proinflammatory cytokines in the liver (A-D), serum (E-H), and hippocampus (I-L), such as tumor necrosis factor-alpha (TNF-α, A, E, I); interleukin beta (IL-1β, B, F, J); IL-6 (DC, G, K); and IL-18 (D, H, L). *, p < 0.05; **, p < 0.01; ***, p < 0.001; NS, not significant. Figure S3. Intranasally administered hiPSC-NSC-EVs incorporated into NeuN + neurons and in multiple brain regions of lipopolysaccharide (LPS) treated mice. A-L: Images illustrating the incorporation of EVs into NeuN+ neurons in the medial prefrontal cortex (mPFC; A, B), the somatosensory cortex (SSC; C, D), dentate granule cell layer (GCL; E, F), the CA1 cell layer (G, H), the CA3 cell layer (I, J) and midbrain (MB; K, L) in LPS-treated mice 6 hours post-administration. The images in B, D, F, H, J, and L represent the magnified versions of images A, C, E, G, I, and K indicated in boxes. Scale bar-A-L, 12.5 μm. Figure S4. Intranasally administered hiPSC-NSC-EVs incorporated into IBA-1+ microglia in multiple brain regions of lipopolysaccharide (LPS) treated mice. A-L: Images illustrating the incorporation of EVs into IBA-1+ microglia in the medial prefrontal cortex (mPFC; A, B), the somatosensory cortex (SSC; C, D), dentate granule cell layer (GCL; E, F), the CA1 cell layer (G, H), the CA3 cell layer (I, J) and midbrain (MB; K, L) in LPS-treated mice 6 hours post-administration. The images in B, D, F, H, J, and L represent the magnified versions of images A, C, E, G, I, and K indicated in boxes. Scale bar-A-L, 12.5 μ [file 12974_2023_2971_MOESM1_ESM.docx]
